# Clustering and Stochastic Simulation Optimization for Outpatient Chemotherapy Appointment Planning and Scheduling

**DOI:** 10.3390/ijerph192315539

**Published:** 2022-11-23

**Authors:** Majed Hadid, Adel Elomri, Regina Padmanabhan, Laoucine Kerbache, Oualid Jouini, Abdelfatteh El Omri, Amir Nounou, Anas Hamad

**Affiliations:** 1 College of Science and Engineering, Hamad bin Khalifa University, Doha 34110, Qatar; 2 Laboratoire Génie Industriel, Université Paris-Saclay, Centrale Supélec, Gif-sur-Yvette, 91190 Paris, France; 3 Surgical Research Section, Department of Surgery, Hamad Medical Corporation, Doha 3050, Qatar; 4 Pharmacy Department, National Center for Cancer Care & Research, Hamad Medical Corporation, Doha 3050, Qatar

**Keywords:** outpatient chemotherapy, cancer, oncology health care, clustering, stochastic simulation-based optimization, multi objectives, planning, scheduling, decision-making metaheuristics, artificial intelligence

## Abstract

Outpatient Chemotherapy Appointment (OCA) planning and scheduling is a process of distributing appointments to available days and times to be handled by various resources through a multi-stage process. Proper OCAs planning and scheduling results in minimizing the length of stay of patients and staff overtime. The integrated consideration of the available capacity, resources planning, scheduling policy, drug preparation requirements, and resources-to-patients assignment can improve the Outpatient Chemotherapy Process’s (OCP’s) overall performance due to interdependencies. However, developing a comprehensive and stochastic decision support system in the OCP environment is complex. Thus, the multi-stages of OCP, stochastic durations, probability of uncertain events occurrence, patterns of patient arrivals, acuity levels of nurses, demand variety, and complex patient pathways are rarely addressed together. Therefore, this paper proposes a clustering and stochastic optimization methodology to handle the various challenges of OCA planning and scheduling. A Stochastic Discrete Simulation-Based Multi-Objective Optimization (SDSMO) model is developed and linked to clustering algorithms using an iterative sequential approach. The experimental results indicate the positive effect of clustering similar appointments on the performance measures and the computational time. The developed cluster-based stochastic optimization approaches showed superior performance compared with baseline and sequencing heuristics using data from a real Outpatient Chemotherapy Center (OCC).

## 1. Introduction

Cancer treatment is a significant healthcare challenge. More than 50% of global cancer cases required chemotherapy in 2018 [1]. Outpatient Chemotherapy Centers (OCCs) worldwide are struggling to satisfy the increasing demand [2,3]. The service quality level and costs of the Outpatient Chemotherapy Process (OCP) are affected by the treatment protocols variety, diverse patient pathways, uncertain durations of services, early and late patient arrivals to the appointments, stochastic patient health conditions, and resource capacities [4]. Thus, the complex OCP that operates in a dynamic environment requires practical and low-cost solutions to increase its efficiency and effectiveness by solving operational issues through superior appointment planning and scheduling.

OCP involves many decisions that must be taken on the strategic, tactical, and operational levels [5]. The strategic decisions determine the number of human (receptionists, nurses, lab technicians, doctors, pharmacists, pharmacy technicians, and aids) and physical (beds, examination rooms, and chemo hoods) resources.

On the tactical level, the primary decision is to use the next-day (split) or same-day scheduling policy. In the split scheduling policy, the patient performs the blood test, and the oncologist reviews the patient health condition and activates the drug preparation order before the appointment day. Therefore, advance drug preparation is applicable in the split scheduling policy, and the nurse directly administrates the drug to the patient on the appointment day. On the other hand, all these processes are performed in one day if the same-day scheduling policy is applied.

The operational level consists of gathering the previous two to operate the OCP efficiently. This level can consist of two stages: first, the days and times of patient appointments are set; second, the human and physical resources are assigned to the patients. The performance of the solutions to the strategic, tactical, and operational issues are assessed by multiple measures. The cost comprises the sum of the salaries and overtime of human resources and the price and operational costs of the physical resources. Each resource utilization is measured by the percentage of actual working time over the available time. The time measures include appointment makespan and delays. The makespan is the time from patient arrival to discharge. The delays are the sum of the mean waiting time, including delay to the first appointment, waiting during the appointment day, and the difference between the planned and actual completion day of all cycles.

OCP represents a complex, multi-stage, multi-server environment. Optimization studies use various idealized assumptions to properly formulate the Outpatient Chemotherapy Appointment (OCA) planning and scheduling problem. Therefore, the majority of the planning and scheduling optimization models considered the optimization of a single stage in OCP. On the other hand, the models that consider multiple stages are deterministic and do not provide a convenient mathematical representation of the stochastic OCP. In addition, they are complex to solve analytically and require long computational times [6,7]. To overcome these challenges, scholars have attempted to develop different heuristics. However, these heuristics could not provide high-quality solutions and are hard to understand and apply in the OCCs.

This contribution proposes using clustering with the Stochastic Discrete Simulation-Based Multi-Objective Optimization (SDSMO) model in a new approach that merges the clustering algorithms with stochastic optimization. The SDSMO uses computer code and simulation to model the objectives and constraints functions of the OCP problems and utilizes patient and appointment data to cope with integrated and enhanced planning and scheduling decisions in uncertain scenarios. The approach clusters similar appointments to reduce the effect of stochastic durations and unpunctual patient arrivals on the schedule. The clustering results are then fed to the optimization model to reduce the computational time.

In the experimental study, various clustering algorithms were linked to the SDSMO model and tested. The SDSMO model is configured to provide solutions to the OCA planning and scheduling problem using an artificial intelligence-enabled general-purpose optimizer. The experiment study used data from a real OCC to compare the performance of the proposed approach that uses clustering to the results of the SDSMO model without clustering, heuristics, and baseline schedules. The following section reviews the current OCP optimization and simulation literature state and shows the necessity of this research for OCA planning and scheduling.

## 2. Literature Review

This review aims to highlight the complexities and uncertainties in OCP, reveal research gaps in the OCA planning and scheduling problem, and propose solutions. The number of studies that have used simulation and optimization in this context is small compared with optimization or simulation only.

Several optimization model types and solving methods are used to handle complexities and uncertainties in OCP. Deterministic optimization models are the most used type of models for OCA planning and scheduling.

The scholars in this area have built upon optimization studies that preceded their work to develop more comprehensive optimization models. Most deterministic optimization models are integer [8,9,10,11,12,13,14,15,16,17,18,19,20,21,22,23,24] or mixed integer programming [7,25,26,27,28,29,30,31,32,33,34]. In mixed integer models, some of the decision variables (but not all) all required to have integer values. Constraint programming was used by [6].

Contrastingly, few optimization studies have proposed stochastic optimization models. A two-stage stochastic integer programming formulation considering the infusion stage only have been developed in [35]. In similar works, the authors of [36,37,38] used stochastic integer programming models and heuristics.

Different simulation models have been used to address several OCP challenges. Scholars focused on using discrete event simulation to improve OCA planning and scheduling [39,40,41,42,43,44,45,46,47,48,49,50,51]. Furthermore, scenario-based analysis has been used to study the effect of capacity planning and scheduling sequencing heuristics on overtime and waiting time [52].

System dynamics simulation models were used to simulate different OCA planning issues in [30,53,54,55]. OCP design and coordination challenges were addressed using agent-based simulation [56,57,58].

Studies containing optimization and simulation models used the simulation model to generate inputs for the optimization model or evaluate output solutions. A chain of articles used both optimization and simulation to study the OCA planning and scheduling problem. The articles [59,60] triggered two research paths that use simulation to evaluate optimization solutions and analyze the OCP, each with several research sub-paths. A one-objective simulation-based optimization to plan shifts of nurses was used in [60].

Different optimization heuristics and exact solution methods were evaluated using simulation [61,62,63,64,65,66]. The authors of [67] developed appointment scheduling rules and compared them using a simulation model. In [68], the simulation model was used to mimic the complexities and uncertainties of OCP. Uncertainty events were generated by a simulation model and used as input to the optimization model in [69].

**Figure 1:**
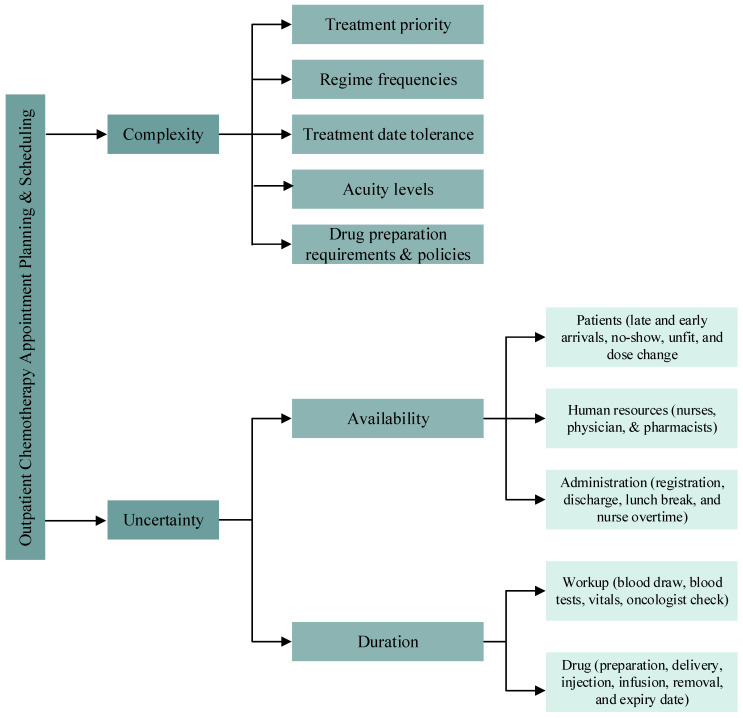
OCP complexities and uncertainties.

Based on the analysis of the reviewed articles, the challenge of the OCA planning and scheduling problem is to account simultaneously for the various complexities and uncertainties shown in Figure 1. This includes the multi-stage processing during infusion because the medical staff is not permanently required. For example, the nurse installs the patient and prepares the infusion, and she can then leave the patient for some time, during which she serves other patients. The flexibility of this work process is known to be hard to analyze because of the complexity of the involved stochastic programming mathematical models. Furthermore, the oncologist can propose tolerances for treatment appointment dates, i.e., a time window for the treatment day. Delaying or bringing forward an appointment within the time window does not affect the health of the patient but also increases the planning flexibility. Again, this flexibility is an additional source of complexity.

On the other hand, an example of uncertainties is the unpunctuality of patient arrivals. Another source of uncertainty is the health condition of the patient at the date of treatment, i.e., the health condition of the patient is not suitable for the prepared drug. These uncertainties are other important features that have significant consequences, given that the prepared drugs may cost several thousands of dollars. Combining the above complexities and uncertainties to enhance OCA planning and scheduling is a challenging task. However, at the same time, it can allow for obtaining insightful results and bridge the gap between the literature and OCP operations in practice.

From the patient pathway perspective, several key performance indicators are studied in the literature to address different issues. The most used performance measures are patient waiting, appointment makespan, and resources overtime. Scholars used different measures for different study scopes. The issues of the OCA planning and scheduling, corresponding performance measures, and the purposes of optimization or simulation models are listed in Table 1. Readers are referred to [5] for more complete references and details about OCP research from an operations management perspective.

### Research Gap and Aim of This Work

In the simulation studies, the existing models often neglected several human resources (Clarks, phlebotomists, and lab technicians) and processes (blood test, vitals measurement, drug order activation, and drip removal) even though they are necessary to the OCP. One of the reasons behind this gap is the use of these simplifications to ease the studies as the OCP problems, such as planning and scheduling, are already complex. Another reason might be the lack of familiarity with different operation models of OCCs. Furthermore, most studies did not show how the models were developed in the used simulation software.

Regarding the optimization and simulation studies, the developed solutions consider limited integration of optimization and simulation models. The simulation models are used mainly to generate data and evaluate optimization solutions. In addition, these studies neglected many of the performance measures that exist in the literature [4].

Table 2 summarizes the studied characteristics of the problem encountered in the literature via a literature synthesis using detailed comparison and indicates how the proposed problem and solution methodology differs from earlier research. A study that used a similar technique was conducted by [19], where the authors used clustering algorithms to cluster similar appointments in small groups (approximately one to seven patients per cluster) and assigned resources to the resulting clusters to solve the one-day OCA scheduling problem. The authors considered one process of the OCP (the drug infusion). Therefore, they clustered appointments based on two infusion-related features, namely infusion duration and acuity level. The clusters of patients were then used instead of individual patients in a modified version of the model from [10] to assign nurses, chairs, and time slots; this optimization model is a deterministic zero-one linear programming model. The binary variables in this model made it difficult to solve. Therefore, [19] introduced clustering to reduce the number of binary variables and thus the computational time. They evaluated their approach using a single performance measure, the patient treatment makespan.

Our study differs from the previous one in several fundamental points: First, we clustered similar appointments in the planning horizon days with consideration of the tolerance limits of the appointment target day. Hence, our clustering algorithm is on the planning level rather than scheduling (appointments of the same day). Furthermore, we considered features related to the drug preparation stage as well as drug infusion, namely the number of drugs to be prepared, eligibility for advance drug preparations, and total drug infusion duration. Therefore, our study analyzed the effect of clustering on all OCP stages rather than the drug infusion stage only. Secondly, our optimization approach is completely different from the single-objective deterministic model in [19]. We used the SDSMO model that explicitly considers uncertainty about assigning patients to days and time slots. Therefore, our work falls under the category of stochastic OCA planning and scheduling. However, different from the single-stage models in [35,36,37,38,64], we considered the multi-stage process, including the patient registration, triage, blood sample extraction, blood test, health condition review, discharge, as well as drug order activation, validation, preparation, delivery, and administration. Moreover, we took into account the different required human and physical resources of each stage, not only the nurses and chairs. As opposed to the mentioned five studies, we did not assume a punctual arrival of patients to appointments. Furthermore, we considered the different patterns of patient arrivals based on their assigned time slots.

On the other hand, same as the model in [36], our model does not decide on patient-to-resource assignments. Nevertheless, we used the acuity levels of patients to restrict the simultaneous nurse tasks. The models in [35,36,37] used a positive integer variable to decide the appointment time without time slots, while the models in [38,64] assigned patients to time slots using a binary variable. We combined both techniques by using discrete variables and restricting their values to a set of feasible values associated with the time slots in the planning days.

The models in [37,64] were solved to optimality using optimization solvers. In contrast, heuristics were used in [35,36,38] to find approximate solutions within a reasonable computational time. We searched for solutions by utilizing an artificial intelligence-enabled General-Purpose Optimizer (GPO) developed by OptQuest. Scholars used GPOs in various other NP-hard operations and logistics applications [70,71,72]. However, GPOs have never been applied to OCA planning and scheduling [5]. Therefore, this work is an extension of this research stream and applies an artificial intelligence-enabled GPO to the OCA planning and scheduling problem for the first time considering the specificities of the OCP problem. This work paves the way for scholars in this research direction by answering the following questions:

- How to develop the SDSMO model for OCA planning and scheduling considering the process of multi-stages, stochastic durations, probability of uncertain events occurrence, performance measures, patterns of patient arrivals, acuity level, demand variety, drug preparation policy, and diversity in patient pathways?
- How to use this model to find solutions by a GPO?
- What is the effect of clustering OCAs using their drug preparation and infusion features on the value of the performance measures and the computational time of stochastic optimization?
- What is the efficiency of the proposed approaches compared with heuristics and real baseline schedules?

## 3. Problem Description 

In this section, the OCP setup and the OCA problem are described. In addition, the baseline approach for OCA planning and scheduling in a real OCC is explained.

### 3.1. OCP Setup

The proposed OCA planning and scheduling approaches are based on a real OCP setup in a large OCC in the gulf region. In the beginning, the primary oncologist confirms the patient cancer diagnosis. Then, the primary oncologist plans the treatment types and sequence based on various examinations such as CT, MRI, and biopsy. For instance, a patient may have to undergo surgery or/and radiotherapy before the start of chemotherapy.

The patient journey in the OCP starts after the primary oncologist determines the chemo protocol and treatment plan. The protocol contains information about the drugs, the number of cycles, durations of cycles, and recovery time between cycles with tolerance limits. The Day Care Unit (DCU) receptionists use the information in the protocol to book the days and times of the OCA. The DCU is the place where the infusion rooms are located. 

Two parallel main stages start before each cycle/appointment, namely, patient health check and drug order preparation. The patient health status needs to be examined before every treatment appointment. Therefore, blood tests and other pretreatment examinations are required.

Depending on the treatment protocol, a patient might take several drugs in one appointment. For each drug, a separate drug order is placed in the system. The oncologists in the DCU review the results and the general health condition of the patient, including weight and height. After that, they review the chemo drug orders and make any required modifications. For example, the drug doses might require modification due to changes in the patient weight. The next step is to approve the drug orders and activate them.

At that point, the preparation process of the drugs starts in the pharmacy. First, the pharmacists perform clinical verification of the drug orders. In the first cycle, the pharmacists review the protocol reference and approvals by the health organization and the scientific literature. Then the blood test results and the dosing weight are checked. In case of required modifications, the pharmacy sends a request to the DCU and waits until the drug order is modified on the system. The second step is adding labels and medication materials to the preparation kit. Then the kit is handed to the pharmacy technician, who prepares the drug and provides it to the pharmacist for the final check. The same steps are repeated for all drug orders of an appointment.

Next, the pharmacy aid transports the prepared drugs to the door of the DCU. The nurses take the drugs and administer them to the patient one by one. The health of the patient is observed after the drug administration is completed. Then the patient is discharged and comes to his next appointment after a specified recovery period.

### 3.2. Baseline Approach

The baseline approach is the current decisions and procedures the case study center applies to handle the OCA planning and scheduling problem in the OCP step described in the previous section. The performance of the baseline approach is compared with the cluster-based SDSMO approach to evaluate the difference in performance in the real environment.

For the human and physical resources dimensioning decisions, the number of human resources depends on the maximum possible number of physical resources. For instance, one room can take a maximum of five beds. Therefore, two nurses are hired to serve each room, considering the acuity levels.

A hybrid scheduling policy is applied, and the blood test follows the split scheduling method. At the same time, the review of results, drug order activation, and drug preparation follow mainly the same-day scheduling policy. Most patients perform their blood tests before the day of the appointment. However, a maximum of one-quarter of patients have their blood tests and health condition reviewed before the appointment. Therefore, the drug orders are activated for only those checked patients. Then their drug is prepared in advance. The rest of the patients are checked on the same appointment day. These patients wait on the beds during the activation and preparation of the drug orders.

The time of patient arrival might be scheduled at 7:00 or 11:00 a.m. considering three-time slots: 7:00–11:00, 11:00–15:00, or 7:00–15:00. An electronic calendar is used to book the appointment days and arrival times manually. For example, an appointment might be planned on Sunday, and the arrival of the patient is scheduled at 7:00 a.m. It is assumed that the patient had his blood test performed one day before. Therefore, the appointment duration is estimated to be the sum of times required for patient registration, health condition review, as well as drug order activation, preparation, delivery, and administration. If the estimated appointment time exceeds 4 h, the patient is assigned to the most extended time slot (7:00–15:00).

The as-soon-as-possible method is applied to plan appointments on days. Nevertheless, the appointments are distributed on the weekdays to have a balanced daily workload considering the appointment day tolerance limits and the drug infusion duration. Suppose that the number of appointments in a certain week (5 working days) is around 250 patients. The daily schedule for this week has around 45 to 55 appointments. 

The patient-to-nurse assignment is decided on the appointment day. The charge nurse attempts to balance the workload among nurses by assigning patients according to their estimated drug infusion duration.

### 3.3. SDSMO Formulation

This section describes the proposed SDSMO formulation of the OCA planning and scheduling problem, driven by the OCP setup and baseline approach presented in the previous sections. There are three main components of the SDSMO model, namely optimization formulation structure, stochastic simulation model (Section 4.1), and optimization methodology (Section 4.2). We next describe and explain the optimization problem formulation. 

In stochastic simulation-based optimization, the simulation model codes replace parts of the optimization formulation to describe the actual process complexity and incorporate its stochastic behavior [73]. The uncertainty in the arrival of patients, durations, and demand of stages is represented using scenarios (simulation replications). A random number generator generates the value of durations and events occurrence of a scenario based on distribution functions and probabilities inserted in the simulation model [74]. Assuming these scenarios and considering that the objective and constraint functions have stochastic parameters, we used the simulation model to measure the objective function and model the constraints. Therefore, in simulation optimization, the optimization formulation consists of the measured objective function using the simulation outputs and the restrictions on the values of the decision variables only [75]. The problem constraints (e.g., capacities, queue disciplines, order of stages, sequence of activities, priorities, and policies) are defined in the simulation model to replicate the real-world process and requirements accurately. Previous studies showed the ability of this approach to find good solutions for planning and scheduling problems in outpatient [76,77,78,79], surgery [80], and emergency [81] departments.

The problem considered in this paper is the planning and scheduling of the patient arrival times (i.e., determining the day and time of patient arrivals) to the chemotherapy appointments where the actual patient arrivals, service times, and stages that are performed during the appointment are stochastic variables. The notations for the optimization formulation are listed below.
Indices:iAppointment; zObjective;dDay in the planning horizon.Sets:IAppointments;OObjectives;SFeasible values of $x_{i},$ start time of appointment slots in the time horizon.Parameters:$N_{I}$Number of appointments;$N_{O}$Number of objectives;$N_{D}$Number of days in the planning horizon;$W_{z}$Weight of objective z∈O;$LB_{i}$Lower bound of planned patient arrival time $x_{i}$ of appointment i∈I;$UB_{i}$Upper bound of planned patient arrival time $x_{i}$ of appointment i∈I;ETEnd time of the planning horizon.Decision variables:$x_{i}$Planned patient arrival time of appointment $i=1,\;2,\ldots ,\;N_{I}$.

The objective is to determine the day and time of patient arrivals that minimizes the following function, which represents the expected total cost of the objective functions.

(1) $$\forall x\in {\mathbb{R}}^{N_{I}}\;\underset{x}{\min}F\left(x\right)=\sum_{z=1}^{N_{O}}W_{z}E[f_{z}\left(x\right)]\;\;W_{z}>0$$

subject to

(2) $$LB_{i}\leq x_{i}\leq UB_{i}\;\;\forall i\in I$$

(3) $$x_{i}\in S\;\;\forall i\in I$$

where $x=\left(x_{1},\;x_{2},\;\ldots ,\;x_{N_{I}}\right)\in {\mathbb{R}}^{N_{I}}$ is the decision vector. The components of the decision vector x are the planned patient arrival times ($x_{i}$) of the appointments in I. The decision vector, x, belongs to ${\mathbb{R}}^{N_{I}}$ ($N_{I}$-dimensional real space), where $N_{I}$ is equivalent to the number of decision variables. 

Since conflicting objectives, $O={\left\{z_{i}\right\}}_{1,N_{O}}$, exist it is impossible to find a single Pareto optimal solution that optimizes all the involved objective functions [82]. Therefore, a single-objective function in (1) is formulated by scalarizing and combining the conflicting objectives, $f_{z}$, using weights, $W_{z}$. In this study, the weights of scalarization are based on expert opinions.

**Figure 2:**
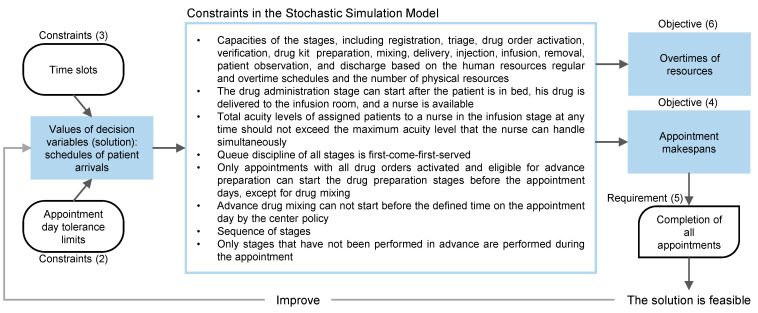
Formulation of the studied OCA problem.

As shown in Figure 2, the values of decision variables are restricted by two constraints to exclude infeasible solutions. Constraints (1) ensure that the value of the decision variables $x_{i}$ ∀i∈I are between the lower, $LB_{i}$, and upper, $UB_{i}$, bounds. The $LB_{i}$ and $UB_{i}$ are determined based on the appointment day tolerance limits. Each appointment has a target day and tolerance limits based on the treatment plan. The scheduling policy defines the start time of the time slots, S, of these days in the planning horizon. Constraints (2) ensure that the values assigned to the decision variables belong to S. For instance, if the target day of the appointment i is Monday with ±1 day tolerance limits, the arrival of the patient, $x_{i}$, can be planned in any time slot on Monday, Sunday, or Tuesday defined in S. In this example, $LB_{i}$ is the first time slot on Sunday, and $UB_{i}$ is the last time slot on Tuesday.

Then, the generated set of values for decision variables is analyzed using the simulation model. As described in Figure 2, the other constraints of the problem are intrinsically incorporated into the stochastic simulation model, including acuity levels, resource capacities, availability, patient availability, priority, queuing discipline, sequence of stages, patient flow, and drug preparation policy.

This study considers two objective functions to test the SDSMO model. The average makespan is the first objective function. $f_{1}$ is the sum of the makespan of all appointments, $MP_{i}$, i∈I over their number,$\;N_{I}$. $MP_{i}$ is measured from the time of actual patient arrival to discharge. This is defined by (4) and calculated by the simulation model. As illustrated in Figure 2, the requirement of demand fulfillment of all appointments is checked by (5) based on the actual patient arrival time, $XA_{i}$, obtained from the simulation model and the value of $MP_{i}$. If (5) is not true for any appointment i, the solution is considered infeasible and excluded. Otherwise, the solution is improved using the methodology explained in Section 4.2.

(4) $$\forall x\in {\mathbb{R}}^{N_{I}}\;f_{1}\left(x\right)=\frac{\sum_{i=1}^{N_{I}}MP_{i}\left(x\right)}{N_{I}}$$

(5) $$MP_{i}+XA_{i}\leq ET\;\forall i\in I$$

The second objective function, $f_{2}$, is the sum of the daily overtime of receptionists, $OVR_{d}$; pharmacists, $OVM_{d}$; pharmacy technicians, $OVE_{d}$; and nurses, $OVN_{d}$ in each day d∈D, over the number of days, $N_{D}$. The generic expression is defined by (6). We developed an algorithm inside the simulation model to calculate the average daily overtime of these resources. This algorithm is presented in Section 4.1.

(6) $$\forall x\in {\mathbb{R}}^{N_{I}}\;f_{2}\left(x\right)=\frac{\sum_{d=1}^{N_{D}}OVR_{d}\left(x\right)+OVM_{d}\left(x\right)+OVE_{d}\left(x\right)+OVN_{d}\left(x\right)}{N_{D}}$$

For the cluster-based approach proposed in Section 4.3, the appointment clusters are considered instead of individual appointments. The same formulation applies with i representing a cluster, I is the set of clusters, $N_{I}$ is the number of clusters, $x_{i}$ is the planned patient arrivals of all appointments in the cluster, $LB_{i}$ is the minimum lower bound of $x_{i}$ of the appointments in the cluster, and $UB_{i}$ is the maximum upper bound of $x_{i}$ of the appointments in the cluster.

## 4. Solution Methodology

As it is evident from Section 2 and Section 3, the problem of OCA scheduling under uncertainty was mathematically formulated and solved optimally for only small problems using idealized assumptions. In this section, we develop a SDSMO model of the OCA planning and scheduling problem to generate solutions using a GPO as an alternative approach. First, we describe the development steps of the stochastic simulation model of the SDSMO model. Second, the solutions generation method using a GPO is discussed. Thirdly, we introduce a framework of an iterative sequential approach that uses clustering to reduce the computational time and enhance the solutions of the SDSMO model. The proposed framework can be adapted to other types of stochastic optimization models.

### 4.1. Stochastic Simulation Model of SDSMO

The stochastic simulation model was developed based on the described OCP setup, problem formulation structure in Section 3, and the flowchart of the OCP of the studied center presented in Figure S1 in the Supplementary Materials. We used AnyLogic to develop the simulation model. AnyLogic is a well-established discrete-event simulation software that uses OptQuest for simulation optimization [83]. Table A1 (Appendix A) summarizes the parameters used in the stochastic simulation model. The main development steps of the simulation model, including the modeling of process flow, constraints, logic coding, and validation of the model, are described next.

We split an appointment into two agents for a more accurate calculation of the performance measures. The first agent is the patient agent. A source block generates the patient agent, reads the patient appointment parameters from a database, and stores it in the patient agent. Then the patient agent goes through checks to determine its eligibility for advance drug preparation.

If the patient agent is found eligible, a copy of this agent is generated under the name drug agent and linked to it (we call this copy Drug Eligible for Advance Preparation (DEAP)). Otherwise, this split happens after the triage or the blood test stages (let it be called Drug Prepared on the Same Day (DPSD)). The DEAP goes to the drug preparation stage directly. In contrast, the DPSD waits for the patient agent to complete the registration, triage, blood test, and drug order activation stages if they are not performed before the arrival of the patient.

The DEAP or DPSD is combined with their linked patient agents after going through the drug preparation stage. The patient agent waits for its copy (drug agent, DEAP, or DPSD) to arrive at this combining point and vice-versa. The combined block destroys the copy (DEAP or DPSD) and outputs the patient agent only. Finally, the patient agent continues its journey in the drug administration and discharge stages.

The following technique is used to model patients’ early, on-time, or late arrival patterns. First, we generate the patient agent way before its planned arrival time ($x_{i}$). Then, we keep it waiting (without including this time in the measure of waiting time) until the time of the earliest expected actual arrival, $XD_{ip}$, of the patients who are following the same arrival distribution p. Subsequently, the patient agent is released and interred in another waiting block. The latter uses the arrival distribution p to randomly release the patient agents after waiting an amount of time equal to $XP_{ip}$. We consider the arrival distributions of the two time slots defined by the scheduling policy of the center, where p=1 and p=2 are the arrival distributions of 7:00 a.m. and 11:00 a.m. appointments, respectively.

For example, the historical data showed that patients with 7:00 a.m. appointments might arrive as early as 45 min prior to their appointment, i.e., 6:15 a.m. The pattern of patient arrivals after 6:15 follows an exponential distribution. Therefore, we set the generation time of patient agents for all 7:00 a.m. appointments to 6:15 a.m. The generated patient agents are entered into a delay block. The delay block uses the exponential distribution to assign waiting times to patient agents. When the waiting time of a patient agent is finished, the delay block releases the patient agent. Thus, from a reporting standpoint, the patient agent appears to have just arrived as it leaves the delay block.

We found that the parameter values of arrival distributions are different for each time slot. For instance, the early morning arrival pattern is different than before noon. Therefore, we considered different arrival distributions that can be identified using historical data for each time slot.

The acuity level is a difficult logic to implement in the simulation model. A nurse is assigned to several patients with total acuity levels equal to the maximum level she can handle simultaneously, $LN_{u}$. Then she can serve one of them at a time for premedication, drug injection, removal, or any other uncertain need. We used Algorithm 1 to simulate this procedure. Using this algorithm, the patient agent can only seize the nurse assigned to it, and a nurse is assigned to patient agents with a total acuity level less than $LN_{u}$.
**Algorithm 1** Simulating patient-to-nurse assignment based on acuity levels1:Find the first available nurse (nurse assigned to patient agents with a total acuity level less than the maximum acuity level of the nurse, $LP_{u}$);2:Check if the to-be-assigned patient agent has an acuity level, $LP_{i}$, less than or equal to the access acuity level that the nurse can handle;3:If step 2 is true, assign the patient agent to the available nurse directly before the premedication stage;4:Increase the number of assigned patient agents to the nurse, $LA_{u}$, by the value equal to the acuity level, $LP_{i}$, of the last assigned patient agent;5:After patient agent discharge, decrease the number of assigned patient agents to the nurse by the value equal to the acuity level, $LP_{i}$, of the discharged patient agent;6:Repeat step 1 to 5 for all patient agents

For optimization we considered the two performance measures (i.e., overtime and makespan) that are explained in Section 3.3 and described in (4) and (6). However, we considered forty-two performance measures for post-optimization analysis under five main categories, including appointment makespan, waiting times between stages, overtime, utilization of resources, and advance drug preparation as shown in Table A3 (Appendix B). The first three categories contain the most used performance measures in OCP studies [4]. However, we included new performance measures under each category which have usually been neglected by previous studies due to the exclusion of several stages. For example, since our model includes most OCP stages, we reported the waiting time in all stages. Furthermore, we considered the overtime of receptionists and pharmacy technicians.

On the other hand, studies have rarely addressed the performance measures of utilization and drug advance preparation. We reported the utilization of several main and secondary resources from all stages to understand the effect of appointment admission policies during different time intervals, namely before, during, and after regular working hours. Moreover, since advance drug preparation is one of the most used strategies to reduce waiting time, we deeply analyzed its performance by considering the number of completed tasks of the main preparation steps. For instance, the number of verified drug orders, and prepared drug kits before the specified time to start the advance drug preparation.

**Figure 3:**
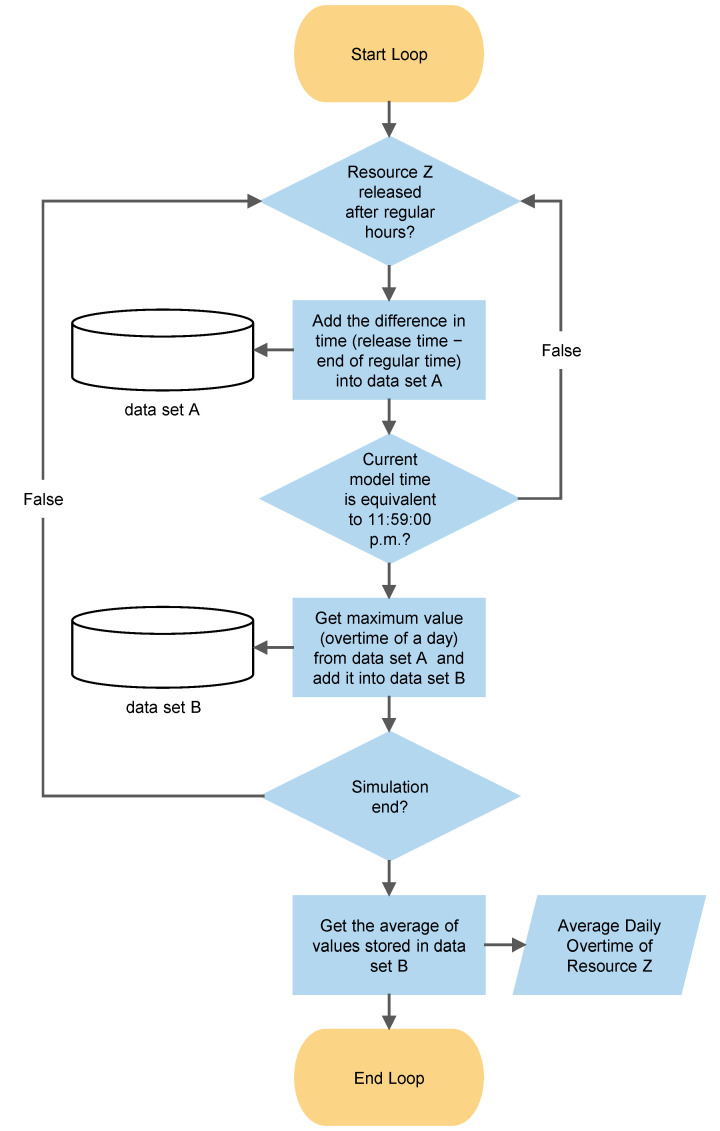
Flowchart of the calculation algorithm of average daily overtime of a resource in the simulation model.

Built-in blocks in AnyLogic can be used to collect performance measures of all categories except overtime of resources. Therefore, we developed the algorithm shown in Figure 3 to calculate the average daily overtime of a resource. In the first step, the time difference between the time of the release of resource Z by a simulated agent and the regular close time of the center is calculated and recorded in data set A. Then the algorithm finds the maximum recorded overtime in data set A and records it in data set B as the overtime of resource Z for that day. After the simulation finishes, the average of values in data set B are reported as the average daily overtime of resource Z.

### 4.2. Solving Method

The decision algorithm and the solution evaluator are the two main components required to generate solutions from the SDSMO model. We used the developed stochastic simulation model as a solution evaluator and the GPO as the decision algorithm. The decision algorithm generates a new set of values for the decision vector, X, at each simulation iteration. A new set is generated by changing the value of one decision variable within a defined feasible range. These values are inserted in the simulation model as input parameters. Then the value of the multi-objective function is calculated based on the simulation results.

The decision algorithm was based on metaheuristics and artificial intelligence used by OptQuest software [84]. Scatter search and tabu search are the two incorporated metaheuristics. The artificial intelligence component uses a multi-layered neural network model [85]. The role of the combined metaheuristics is to guide the decision algorithm toward a new set of values for decision variables. The tabu search uses an adaptive memory to prevent the scatter search from regenerating a previously evaluated set of values and guide the diversification and intensification of the search process.

The neural network is utilized to accelerate the search process by excluding the sets of values that are predicted to produce inferior values of the multi-objective function [86]. In other words, the neural network helps reduce the number of simulations whose results are likely to be poor. The data of the evaluated solutions (values of the decision vector and objective function) are used to train the neural network. The training continues until a prespecified minimum error between the known and predicted objective function values is reached.

The OptQuest algorithm focuses on each objective at the beginning of the search. Then, the successor iterations fill the gaps on the Pareto frontier [87]. Readers are referred to [85] for a broader description of the OptQuest algorithm as well as its formulas.

The OptQuest algorithm writes the values of the decision variables in the simulation model parameters. AnyLogic software runs the simulation model using the parameter values from OptQuest. At the end of the run, AnyLogic exports the simulation results to the OptQuest algorithm. The latter assigns the value of the multi-objective function to the current set of values of decision variables.

The search process continues unless terminated by stopping criteria. The simulation-based optimization literature has two main approaches for defining the stop criteria [88]. The most used approach applies arbitrary criteria specified depending on the running time, the number of consecutive non-improving solutions, or the number of decision variables. The second approach was based on Karush–Kuhn–Tucker conditions that use gradients for the stopping criterion. OptQuest supports the first approach only [89]. The recommended minimum number of simulation iterations for multi-objective optimization with over 100 decision variables is 25,000 [90]. Therefore, we used this recommended number of iterations as a stop criterion to evaluate the performance of the model. For each iteration, the replication stops when the desired confidence level and error percent are reached after a minimum defined number of replicates by the user.

### 4.3. Clustering and Stochastic Optimization Framework

There are two main reasons behind the use of clustering in our solution approach. The first reason is to reduce the effect of the unpunctuality of patient arrivals on the performance of the solutions. In our approach, we take all problem factors, such as the number of time slots and the number of resources, as inputs to the optimization problem and we only decide on the day and time of patient arrivals. Unpunctual arrivals directly affect the performance of the solution. Therefore, we cluster similar appointments to minimize the effect of unpunctual arrivals on the schedule of the appointments.

**Figure 4:**
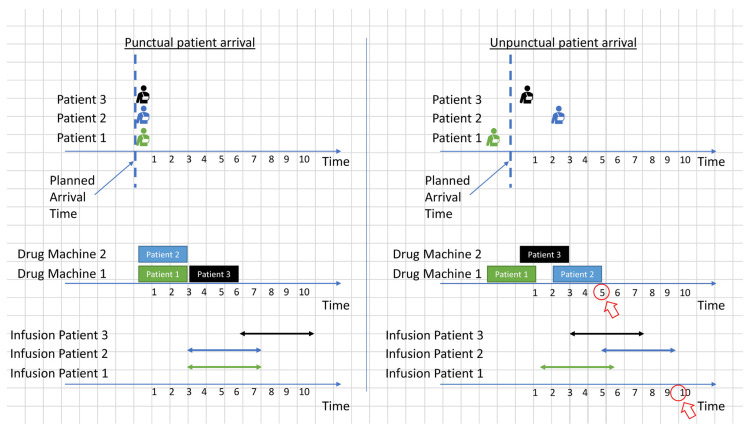
Example of a schedule of clustered appointments with punctual and unpunctual patient arrivals.

**Figure 5:**
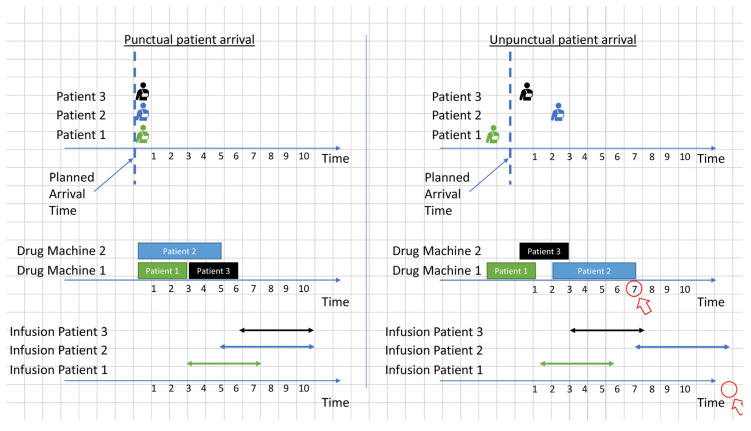
Example of a schedule of non-clustered appointments with punctual and unpunctual patient arrivals.

For example, consider the two scenarios shown in Figure 4 and Figure 5, with clustered and non-clustered appointments. The rectangles show the required time to prepare the drugs of the patient, and the bidirectional arrows represent the infusion time. In these two scenarios, the drug preparation cannot be started before the arrival of the patients. Although the two scenarios have different appointment groups, drug preparation and infusion completion times are the same under the assumption of punctual patient arrivals (left side of Figure 4 and Figure 5). In contrast, when uncertainty in patient arrivals is introduced (right side of Figure 4 and Figure 5), the non-clustered appointments take much longer than the clustered appointments to complete all appointments. This is because the clustered appointments are exchangeable, and the order does not matter when unpunctual patient arrivals disrupt the schedule.

The second reason is to reduce the number of decision variables used in the SDSMO model. This is because using a smaller number of decision variables by clustering appointments decreases the computational time required for the same size of problem. Moreover, this is useful when the SDSMO model is running in a software that specifies a limit on the number of parameters of the simulation model. Therefore, the use of appointment clusters instead of individual appointments allows the SDSMO model to solve problems involving a number of decision variables more than the specified limit.

The proposed framework consists of two main steps, clustering and feeding the clusters to the SDSMO model. First, all received appointment requests are kept in a queue. Then, these appointment requests are entered into a clustering algorithm to generate appointment clusters. For that purpose, we used appointment features related to the appointment time window, the drug preparation stage, and the drug infusion stage. We included the target appointment day to increase the number of clusters that contain similar appointments with the same or close target day. For instance, if the appointment target day is Monday, this appointment is clustered with appointments that have similar features on Monday or appointments of the closest day to Monday. Consequently, fewer possible values of the decision variable (i.e., planned arrival time) are considered. This is because the distance between the lower and upper bounds is reduced.

The other features are used to cluster similar appointments that are exchangeable at the different stages of the process, including drug order activation, preparation, and infusion. For example, the drugs of the clustered appointments in Figure 4 are all ineligible for advance preparation and have the same durations of drug preparation and infusion. Finally, the obtained appointment clusters are fed to the SDSMO model for day and time assignment.

We considered testing different clustering algorithms in the experimental study. K-means was used as it is one of the classic algorithms available. Furthermore, we also considered Hierarchical and Self-Organizing Maps clustering. Our intent is not to compare these clustering algorithms, but we used them to verify the proposed framework.

**Figure 6:**
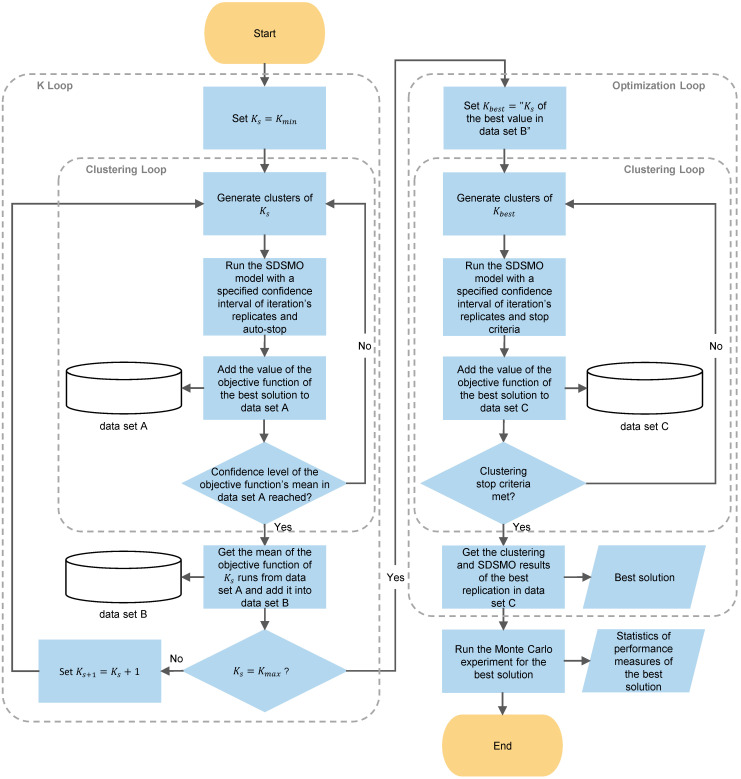
The proposed iterative sequential approach to link clustering to stochastic optimization.

We propose the methodology shown in Figure 6 to link the clustering algorithms to the SDSMO model. As opposed to [19] we do not recommend a guideline to choose K because we think just saying K equals the number of patients over the number of nurses plus one is not enough to justify the choice. Furthermore, this kind of clustering algorithm belongs to unsupervised learning that provides different K clusters in each run due to its random start. Therefore, there should be a criterion to choose the clustering results of the chosen K. We used a maximum number of clustering replications same as the work in [19]. However, we did not pick the clustering results of K based on the lowest summation of distances between points and their cluster centroids.

In our iterative sequential approach, we used the clustering and optimization loops illustrated in Figure 6 to choose the best value of K and clustering results. First, in the K loop, the $K_{s}$ of the first round, s=1, is set to $K_{min}$. We chose $K_{min}$ be equal to the number of daily time slots multiplied by the number of planning days. This value of $K_{min}$ was chosen to have at least one cluster for each time slot in the planning horizon and let the SDSMO model decides the distribution of these clusters on the time slots. In the clustering loop, the clustering algorithm was used to generate $K_{s}$ clusters. Then, the clusters are fed to the SDSMO model to obtain the value of the objective function. To reduce the time required to complete the optimization experiment, we used less strict search stop criteria by enabling the automatic stop option. This means that the optimization experiment stops when the solution is not improving after OptQuest has used all its techniques for the default number of times (two) specified by AnyLogic [91]. However, we maintained a 95% confidence level and 5% error with a minimum of two replicates as a stop criterion for simulation replications of a solution iteration.

The value of the objective function is added to data set A, and the same steps are repeated for the same $K_{s}$ until the 95% confidence level with a 5% error of the mean value of the objective function is reached. The mean of the objective function values of $K_{s}$ is stored in data set B. After that, $K_{s}$ value is increased by one, and the loop is executed again until $K_{s}=K_{max}$. We chose $K_{max}$ to be equal to the number of appointments divided by two. Since each cluster has one decision variable in the SDSMO model, the chosen value of $K_{max}$ ensures that the number of decision variables is reduced by half at least. This allows for reaching the minimum desired reduction in the computational time. Finally, the $K_{s}$ that corresponds to the best mean value of the objective function in data set B is chosen as $K_{best}$ for the optimization loop.

The same clustering loop in the K loop is used in the optimization loop with different stop criteria. First, for the SDSMO model, we used a specified number of maximum simulation iterations (e.g., 25,000 [90]) to be conducted instead of an automatic stop. Since running the SDSMO model for a large number of iterations takes a relatively long time, we chose to terminate the clustering loop after the 95% confidence level with a 5% error of the mean value of the objective function is reached, or a maximum number of clustering replications (e.g., seven [19]) of $K_{best}$ is completed. Then, the clustering and SDSMO results that correspond to the best objective function in data set C was chosen as the best solution. Finally, we took this solution and ran a greater number of replications using the Monte Carlo experiment to estimate the values of the objective and performance measures with a smaller margin of error.

## 5. Experimental Design

In this section, we demonstrate the design steps of a comprehensive computational study that is used to evaluate the solution methodology. First, we validate the reliability of the developed stochastic simulation model in representing the real OCP. Second, the verification procedure of the proposed approach is presented.

### 5.1. Validation of the Stochastic Simulation Model

A senior pharmacy informatics officer collected empirical operations data from a large OCC in the gulf region. Appointment and process data over three months were extracted from the deployed healthcare information system in the center. The data included time stamps at the beginning and end of each major stage and service of all appointments. In addition to actual and scheduled patient arrivals and drug order data.

We analyzed the data using a statistics package (Minitab) to determine the best-fit duration distributions of stages and probabilities of stochastic parameters. We used the *p*-value of the Anderson–Darling Test (ADT) to justify the distribution choices. If an optional extra parameter was added in a distribution, we used the *p*-value of the Likelihood-Ratio Test (LRT) to indicate the importance of adding the extra parameter. For example, in Table A2 (Appendix A) the duration of the registration stage was found to be following a Weibull distribution, the *p*-value of ADT is greater than 0.005, and the *p*-value of the LRT is less than 0.005, which indicates the adequacy of the distribution and the importance of adding the third parameter [92,93]. As shown in Table A2 (Appendix A), expert opinion and literature were used when data were unavailable to generate portability distributions. Consequently, some values of the stochastic parameters were calculated using deterministic functions for experimental purposes.

We used historical data and medical and management staff feedback to validate the simulation model. Length of stay is the only reported performance measure in the collected data. Therefore, we compared the average makespan from the historical data with the average makespan measured by the stochastic simulation model using the baseline schedules.

Our simulation model contains a large number of stochastically varied parameters. Therefore, we used the Monte Carlo method because its accuracy is independent of the number of stochastic parameters in the simulated model [94]. We ran 100 simulation replicates using the Monte Carlo experiment in AnyLogic to consider the effect of stochastic parameter values. As shown in Table 3, the 100 simulation replicates allowed the analysis of the makespan with 0.008 half-width (HW) of the 95% confidence interval (CI).

**Figure 7:**
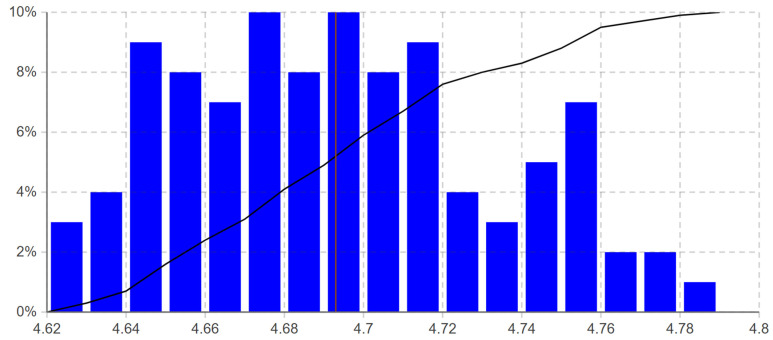
Average makespan distribution of the 100 simulation replicates from the performed Monte Carlo experiment in AnyLogic (The red vertical line is at the mean of the average makespan of the simulation replicates, and the black polyline is the cumulative distribution function).

The histogram shown in Figure 7 visualizes the distribution of the average makespan of the 100 simulation replicates. The minimum and maximum obtained average makespan values and the deviation are 4.628, 4.784, and 0.04, respectively. The results in Table 3 and Figure 7 indicate that the simulation model is a tractable representation of the actual process. Moreover, the staff feedback confirmed the reliability of the simulation model. The system data and expert opinion helped to represent the process accurately.

### 5.2. Verification of Clustering and Stochastic Optimization Framework

As shown in Table 4, the experimental design included four experiment sets to be used to answer the research questions and verify the proposed approach. The first set investigates the impact of clustering on the performance of the SDSMO model and the solution.

We conducted the experiments of the first set using the two sets of clustering features shown in Table 5. Set A has four features, and Set B includes the same features except one feature related to the eligibility of advance drug preparation. These features were selected based on the reasons explained in Section 4.3. We excluded the feature of eligibility for advance preparation in the second set because the percentage of eligible drug orders that are actually prepared in advance is variable. Therefore, we analyzed the effect of including and excluding this feature on the exchangeability of drug orders at the drug preparation stage. Our aim is not to find the optimal combination of features. We used these two sets as examples to show the effect of the used features on the proposed approach.

The results of the first sets were compared with the solution of the SDSMO model without the use of clustering to evaluate the effect of clustering on the computation time and the value of the objective function. Furthermore, we used the third set to perform an objective comparison with sequencing heuristics from the literature [52,95]. As benchmarks, we used nine different sequencing heuristics based on the eligibility for advance drug preparation (expensive and not expensive drugs) and durations of drug preparation and infusion. Moreover, the baseline schedules were used in the fourth set to assess the value of the proposed approach in practice.

One-week scenarios were used to calculate the value of the multi-objective function and the performance measures. This week had 246 appointments distributed over five days with the drug and patient data listed in Table S1. The value of the clustering features of sets A and B are in Table S2. Tables S1 and S2 are available in the Supplementary Materials of this paper.

The resource data that were used in the experiments are shown in Table 6. As can be seen, some resources work an extra amount of time before and after the regular hours. The overtime before the regular opening time is one hour for all overtime resource types. On the other hand, overtime resources work after the regular hours for varying amounts of time that depend on the required time to complete all tasks.

As explained in Section 4.2, we ran the simulation-based optimization experiment for approaches 1.1–2.1 until the stop criterion was met. We used 25,000 maximum number of iterations as suggested in [90] for problems with more than 100 decision variables to stop the experiment. The behavior of the tested solution under stochasticity was considered by using a varying number of replications. For each iteration of a solution, the replication stopped when a 95% confidence level and 5% error were reached after a minimum of two replicates. Then, the next iteration was started, and the same replication procedure was repeated.

The obtained solutions of approaches 1.1–2.1, as well as the solutions of the sequencing heuristics and the baseline schedules (approaches 3.1–4.1) were simulated for 10,000 replications. This number of simulation replicates allows the analysis of the objective function and performance measures with HW in the less than ± 1% range. For that purpose, we used the Monte Carlo experiment in AnyLogic to compare the solutions of approaches under the effect of stochastic parameters.

For each solution, the simulation replicates were run using random values of the stochastic parameters in each run. Finally, we reported the obtained values of the mean, minimum, maximum, standard deviation, and HW of the 95% CI of the value of the objective function, as well as the forty-two performance measures considered in the post-optimization analysis.

## 6. Results and Discussion

In this section, we first determine the set of features for approaches that use clustering (1.1–1.3). After fixing the feature set, we compare the performance of the SDSMO approaches without and with clustering (experiment sets 1 and 2) to assess the value of clustering. After that, we report the percentage gap between the solution found by the first set with sequencing heuristics from the literature. We also compare the solution with the baseline schedules.

Finally, we analyze the results of the Monte Carlo experiment of the forty-two performance measures that are considered for post-optimization analysis to assess the solutions in practice from various perspectives. The experiments were all performed on H.P. Pavilion Gaming Laptop with an AMD Ryzen 5 5600 H processor running at 3.30 GHz with 32.0 GB RAM.

### 6.1. Determining the Set of Features

In this section, we perform approaches 1.1–1.3 using the two sets of features A and B. In the subsequent sections, we use the set of features that helped achieve better objective functions. As we see next, the set of features is an important parameter, as it directly affects both the solution quality and run time spent to obtain a solution.

We applied the algorithm shown in Figure 6 to obtain the clustering results for the sets of features A and B. We ran the SDSMO model for each clustering result until the specified stop criteria in Section 5.2. Table 7 shows the objective values and the associated run time to obtain the solutions. When set B is used, the objective value improves by more than 17%, 12%, and 7% for approaches 1.1–1.3, respectively. The model run time needed to reach the stop criteria increases significantly when set B is used. Compared with the literature, this run time is short for the considered problem size (Table 2).

Nevertheless, we used the comparison in Table 8 to analyze the reason behind this improvement of objective values and the increase in computational time. We analyzed the number of clusters that have all appointments with the same target day and the number of those that have appointments with different target days. For example, a cluster with appointments of different target days might have three appointments with Monday as the target day and two appointments on Tuesday. When all appointments are on the same day, this means fewer possible values for the decision variable, which is the day and time of arrival of patients.

For instance, considering two-time slots in a day, the possible number of values of decision variables in Approach 1.1 with the set of features B is two only for the majority of the clusters. On the other hand, the number of values of decision variables in Approach 1.1 with the set of features A is two for 75% of the clusters and four for 25% of the clusters (two days with two-time slots each). Therefore, when set B is used, the search space is smaller because fewer possible values of decision variables need to be checked. The decision algorithm concentrates on enhancing the solution of one day instead of exploring the solutions that mix appointments of two days. However, the varying number of required replicates of each iteration to reach the 95% confidence level with an error of 5% that the current solution is better than the last tested one is significantly higher because the difference between the solutions is minimal. Therefore, the computational time increases. Another reason is related to the additional feature used in set A, which is the eligibility of all drugs for advance preparation. This feature has a minor effect on the exchangeability of drug orders at the drug preparation stage. This is because a low percentage of eligible drug orders are actually prepared in advance due to the current applied conservative policy in the center.

Finally, we did not compare the results of the clustering algorithms because rather than the accuracy of clustering (summation of squared distances between the data points and their nearest centroids), it is the features of appointments in each cluster that makes the difference in the planning and scheduling. Particularly, the purpose of the previous comparisons is to indicate that clustering can help reduce the computational time and enhance the solution of the utilized stochastic optimization model, provided the right set of features is used. In the following comparisons, we use the results of Approach 1.1 with the set of features B to report the gap and the difference in computation time.

### 6.2. Comparison of Stochastic Optimization with and without Clustering

To assess the value of appointment clustering with the optimization model, we compared the results of Approach 1.1 with Approach 2.1, which does not use the clustering results. We kept all the settings of the SDSMO model the same as described in the experimental design section.

Based on the objective values and computational times in Table 9, Approach 2.1 spends approximately eight and a half hours more than Approach 1.1 for the same stop criteria. The gap between the mean objective value from Approach 1.1 solution is almost 15%. The results indicate that the proposed clustering methodology for stochastic optimization of the OCA problem helped the SDSMO model find a better solution in considerably less computation time (approximately 48%). The standard deviation and the 95% CI HW of Approach 1.1 are higher than Approach 2.1. This statistically means that clustering improved the accuracy, but also increased the scattering (standard deviation). As a consequence, if one wants more accurate results, clustering should be used; but if one wants less scattered (i.e., more repetitive and predictable), clustering should be avoided.

### 6.3. Benchmarking with Sequencing Heuristics

To justify our claim in Section 4.3 that the use of clustering is an appropriate method to decrease disruption in the schedule of OCAs that is caused by the unpunctuality in the arrival of patients, we compared our clustering-based solution with the solutions of sequencing heuristics from the literature.

In Table 10 we present the percentage gap between the objective value associated with the clustering-based approach 1.1 and the heuristics approaches 3.1–3.9. Based on these results, the clustering solutions can obtain a better objective function value than the benchmark heuristics.

LIDF has the best performance among these heuristics, generating a 12.5% gap with approach 1.1. For EDF and EDLIDF, we observe relatively larger gaps, around 23%. These results indicate that it is important to consider appointment clustering to reduce the effect of stochasticity on the solution. 

### 6.4. Comparison with the Baseline Schedules

We assessed the value of using clustering and the SDSMO model in practice by comparing Approach 1.1 with the baseline schedules (Approach 4.1). The comparison results are shown in Table 11. We compared both approaches with respect to the average objective value, makespan, and overtime. The baseline schedules yielded significantly worse objective values. Approach 1.1 outperforms the baseline in terms of overtime value by almost 18%. Due to the use of only two-time slots in both approaches, the improvement in average makespan value is particularly small.

### 6.5. Analysis of Performance Measures

The previous results are obtained from a complex stochastic simulation model of the OCP queuing network. This network comprises the arrivals, queues, service times, and paths. Therefore, there are three categories of randomness sources in the developed simulation model that are listed as stochastic parameters in Table A1 (Appendix A). The first one is the patterns of patient arrivals, which are defined by arrival distributions, as explained in Section 4.1. The second source of randomness is the service time distributions. Finally, the third source of randomness is the probabilities that decide the paths of patients or drug agents in the process.

We analyzed the effect of these sources of randomness on the forty-two performance measures (described in Section 4.1) that are considered for post-optimization analysis using the Monte Carlo experiment in AnyLogic. The analysis shown in Table A3 (Appendix B) indicates that the overall performance of Approach 1.1 under stochasticity is better than the other approaches listed in Table 5. Approach 1.1 achieved the lowest average overtime and lower average makespan than most approaches. 

Nevertheless, the utilization of the main resources, such as beds, nurses, and pharmacy technicians, is low. This indicates that there is a capacity to have more appointments to be served on the analyzed week. However, the advance drug preparation requirements and policy restrict the use of the remaining capacity. For instance, although around 35% of drug orders were verified and their kits were prepared before 06:00 a.m. (the time to start advance drug production as per the center policy), less than 19% were produced in advance. This is because the remaining drug orders contain expensive drugs. Therefore, the pharmacy waits until the admission of the patient to start the drug production of these orders due to the applied policy.

The comparison of the performance measures indicates that the clustering of appointments and the use of the SDSMO model considerably increased the overall performance of the process. The better patient-to-time slots assignment enhanced the patient flow through the stages. Furthermore, it was possible to decide on more efficient appointments for the scenarios of uncertainties summarized in [5].

Furthermore, the SDSMO model verified its remarkable ability to utilize the scenario characteristics while reacting to uncertainties. All the proposed clustering-based approaches achieved better performance than sequencing heuristics and baseline schedules. Clustering similar appointments had a positive effect on the overall performance of the OCC.

Moreover, this approach helped reduce the schedule disruption caused by unpunctual patient arrivals. This is because the drug preparation tasks of the clustered appointments are exchangeable due to the similarity in their required preparation time. Therefore, clustering the OCAs reduces the effect of the change in the order of drug preparation tasks caused by unpunctuality in patient arrivals on the performance of the overall schedule.

## 7. Limitations and Future Research Directions

There are some limitations of the research in this article. The first limitation is mainly related to the considered scope of the optimization problem. In the problem design, we focused on the planning and scheduling of patient arrivals. Although this strategy facilitated the study of a relatively large problem, considering the resource–patient assignment decisions can help analyze the proposed approach in more depth. Furthermore, we address the planning of appointments within their tolerance days, regardless of the fact that each appointment is one cycle of a series of appointments separated by recovery days.

Additional studies can build on this work by examining the effect of linking patient appointments in the planning horizon on the continuity of care. We used makespan and overtime for the objective function. The study can be extended to include other performance measures, such as fairness and patient preferences. In addition, we defined the problem and developed the SDSMO model based on the literature and the observations and data from one OCC. A generic model based on a wide range of OCC environments is needed to generalize the results.

Second, we used stochastic simulation optimization to model the problem and enhance the solution. This method does not require a complete mathematical formulation of the objective and constraint functions. Furthermore, the deployed metaheuristics can find high-quality solutions. However, it does not guarantee near-optimal or optimal solutions. In addition, we used a user-defined criterion based on the number of decision variables and computational budget to stop the search. An alternative is to use gradient-based stop criteria. Therefore, formulating a multi-stage multi-objective stochastic mathematical model and solving it optimally within a reasonable computational time represents a challenging future research direction.

Third, in the experimental study, we tested three clustering algorithms only. Integrating other clustering algorithms with the optimization models of OCA planning and scheduling problems leads to a more comprehensive analysis. Moreover, the proposed iterative sequential clustering and optimization approach considered only unsupervised clustering algorithms. Semi-supervised and supervised clustering can be used to produce class-uniform clusters, particularly if we want to extend the scope of the paper beyond the OCA problem.

Therefore, this study can be extended to a more in-depth assessment of the proposed approach. The first potential future research direction is to re-evaluate the tradeoffs between different objectives in the multi-objective function and use more objectives. The four classes of multi-objective optimization methods, namely no preference, a priori, a posteriori, and interactive should be used and compared.

Furthermore, a test case in a real environment is required to validate the accuracy of the simulation model. Finally, further studies can implement the proposed approach in an adaptive manner to handle stochastic events such as the inability of patients to take the treatment. Moreover, the full-factorial experimental design can be used to jointly consider the strategic, tactical, and operational decisions to assess their interaction. The comprehensive multi-stage stochastic simulation model developed in this article can be used to fulfill this goal.

Another extension can be related to robotic chemotherapy compounding. By using the iterative sequential clustering and stochastic optimization approach proposed in this research, future work can facilitate the transition from manual to automated drug production. In the automated process, the pharmacy staff loads the robot with the drug order information and raw materials [96]. The main challenge of applying this technology is the number of required robot setups per day [97]. Therefore, appointment clustering and scheduling optimization are needed for batch production of identical drug orders instead of individualized production.

## 8. Conclusions

Appointment planning and scheduling in multi-class open queuing networks with feedback such as the OCP remain challenging, as it comprises many uncertainties, such as stochastic arrivals and service durations. Advance drug preparation before the arrival of patients is a common strategy applied by the OCCs to improve the OCP. However, the number of eligible drug orders for advance preparation that are prepared in advance is small due to several restrictions such as patient health, drug validity period, and unpunctuality in patient arrivals. Hence, there is a need for a planning and scheduling approach that considers the stochasticity and uncertainty in the process while assigning days and time slots to appointments.

Therefore, this research proposes an iterative sequential approach for OCA planning and scheduling. The approach uses clustering algorithms to generate appointment clusters based on the similarity in the target appointment day, drug orders, and infusion durations. Then, a stochastic optimization model is used to decide the days and times of the arrival of patients for these appointment clusters. 

From the conducted experimental study, it is evident that the proposed approach improves the overall performance of the OCP, helps the SDSMO model to find better solutions in less computational time, and enhances the responsiveness of the solution to stochasticity. The proposed approach performs exceptionally well compared with other heuristics and the baseline schedules. Furthermore, the Monte Carlo analysis showed the robustness of the approach using randomly generated values for the stochastic parameters.

This research has several potential extensions for OCA or other appointment planning and scheduling problems. The proposed framework and models can be tested in different environments. Second, a scenario-based analysis using the developed simulation model and algorithms can reveal interesting findings. One interesting future direction would be to coordinate the arrival of patients with automated batch drug production.

## Figures and Tables

**Table 1:** OCP issues and performance measures.

Issues	Measures	Study Scope
Time	Treatment delay	Planning
	Waiting time	Scheduling
	Makespan	
Cost	Overtime	Scheduling
	Number of staff	
	Number of equipment	
	Inventory levels	
Workload	Fairness	Assignment
	Capacity	Planning and scheduling
	Utilization	
Satisfaction	Demand fulfillment	Planning
	Treatment efficiency	Assignment
	Patient preferences	Scheduling

**Table 2:** Previous relevant OCA research.

Reference	Optimization Problem	Objective Function	Improvement Level	Uncertainty	Uncertainty Handling	Model Type	Solving Methods	Problem Size/CPU *
[19]	Scheduling and assignment	Minimize makespan	Single stage: infusion	-	Deterministic	Integer programming	Clustering, and exact	150/852
[35]	Scheduling and assignment	Minimize waiting time, and makespan	Single stage: infusion	Drug infusion duration	Stochastic	Stochastic mixed integer programming	Heuristic	12/18
[36]	Scheduling and assignment	Minimize waiting time, overtime, and chair idle time	Single stage: infusion	Drug infusion duration	Stochastic	Stochastic mixed integer programming	Heuristic	8/30
[37]	Scheduling and assignment	Minimize waiting time and overtime	Single stage: infusion	Drug infusion duration	Stochastic	Stochastic mixed integer programming	Exact	12/6
[38]	Scheduling and assignment	Minimize waiting time, overtime, and excess acuity	Single stage: infusion	Drug infusion duration	Stochastic	Stochastic mixed integer programming	Heuristic	7/6
[52]	Scheduling	Minimize waiting time and overtime	Multistage: registration, triage, drug preparation, drug infusion, and discharge	Patient arrivals	Stochastic	Discrete event simulation	Sequencing heuristics	
[64]	Planning, scheduling, and assignment	Minimize first appointment start delay, and scheduling conflicts	Single stage: infusion	Drug infusion duration, and nurse availability	Stochastic	Mean-risk stochastic integer programming	Exact	
This paper	Planning and scheduling	Minimize makespan and overtime	Multistage: registration, blood draw, triage, oncologist visit, drug preparation, drug infusion, and discharge	Duration of stages and services, patient arrivals, probabilities of patient and drug orders paths	Stochastic	Stochastic discrete simulation-based optimization	Clustering, and Meta-heuristics	246/786

* Number of appointments and approximated computation time (in minutes).

**Table 3:** Comparison of the actual and simulated average makespan of the baseline schedules (in hours) for three months.

Historical Data	Monte Carlo Experiment		
Average Makespan	*n*	Mean	HW of the 95% CI
4.71	100	4.69	0.008

**Table 4:** Experiments sets.

Set	Approach
1.1	SDSMO for planning & scheduling of the K-means clustering results
1.2	SDSMO for planning & scheduling of the Hierarchical clustering results
1.3	SDSMO for planning & scheduling of the Self-Organizing Maps clustering results
2.1	SDSMO for planning & scheduling of individual appointments
3.1	Expensive Drugs First (EDF)
3.2	Expensive Drugs and Long Infusion Duration First (EDLIDF)
3.3	Long Drug Preparation Duration First (LDPDF)
3.4	Short Drug Preparation Duration First (SDPDF)
3.5	Short Infusion Duration First (SIDF)
3.6	Long Infusion Duration First (LIDF)
3.7	Plateau Pattern (P.P.)
3.8	Not Expensive Drugs First (NEDF)
3.9	Not Expensive Drugs Short Infusion Duration First (NEDSIDF)
4.1	Baseline schedules

**Table 5:** The included appointment features in the sets of features A and B.

Appointment Feature	Set A	Set B
Target appointment day	•	•
Number of drugs to be prepared	•	•
Eligibility of all drugs for advance preparation	•	
Total infusion duration of all drugs	•	•

**Table 6:** Resource data.

Resource	Amount	Overtime before Regular Hours (hour)	Overtime after Regular Hours (hour)
G	2		
L	2		
C	2		
$R,R^{\prime },R^{\prime \prime }$	3, 1, 1	1	Until the completion of all tasks
$B,B^{\prime },B^{\prime \prime }$	42, 42, 42	1	
$M,M^{\prime },M^{\prime \prime }$	4, 1, 1	1	
$E,E^{\prime },E^{\prime \prime }$	2, 2, 1	1	
$A,A^{\prime },A^{\prime \prime }$	2, 2, 1	1	
$N,N^{\prime },N^{\prime \prime }$	16, 2, 2	1	

**Table 7:** Objective function values and run times (in hours) of the clustering approaches 1.1–1.3 using two different sets of features and the same stop criteria for the SDSMO model.

Approach	Set of Features A						Set of Features B					
	Objective					Time	Objective					Time
	µ	Min	Max	σ	95% CI HW		µ	Min	Max	σ	95% CI HW	
1.1	402.1	231.4	1475.9	107.5	2.1	4.4	338.2	155.2	1476.1	110.5	2.2	13.1
1.2	404.1	211.1	1272.9	107.0	2.1	3.9	358.2	175.2	1195.9	110.0	2.2	10.9
1.3	375.1	189.5	1204.3	104.2	2.0	5.7	348.4	190.9	1646.5	109.0	2.1	9.8

**Table 8:** Percentages of clusters with appointments that have the same target day and two target days in the clustering results of the sets of features A and B that are used to obtain the results in Table 7.

Approach	Set of Features A		Set of Features B	
	One Day	Two Days	One Day	Two Days
1.1	75	25	98	2
1.2	80	20	96	4
1.3	67	34	93	7

**Table 9:** The gap between the solutions and the run times (in hours) of the SDSMO model with and without clustering.

Result		Approach 2.1	Approach 1.1	Gap (%) ^1^
Objective	µ	392.0	338.2	14.7
	Min	211.4	155.2	30.7
	Max	1588.9	1476.1	7.4
	σ	106.5	110.5	−3.7
	95% CI HW	2.1	2.2	−4.7
Time		21.3	13.1	47.7

^1^ The minus sign before the percentage indicates that the result of Approach 1.1 is higher than the result of Approach 2.1.

**Table 10:** The gap between the solution of the SDSMO model with clustering and the sequencing heuristics.

Result	Objective Value	Gap (%) *								
	Approach 1.1	EDF	EDLIDF	LDPDF	SDPDF	SIDF	LIDF	PP	NEDF	NEDSIDF
µ	338.2	−22.9	−22.4	−18.8	−19.2	−21.9	−12.5	−12.6	−15.6	−15.9
95% CI LB	336.1	−23.1	−22.6	−18.9	−19.3	−22.0	−12.6	−12.7	−15.7	−15.9
95% CI UB	340.4	−22.8	−22.3	−18.7	−19.0	−21.8	−12.4	−12.5	−15.5	−15.8

* The minus sign before the percentage indicates that the result of Approach 1.1 is better than the result of the compared sequencing heuristic.

**Table 11:** The gap between the solution of the SDSMO model with clustering and the baseline schedules.

Results		Objective Value		Makespan		Overtime	
		Approach		Approach		Approach	
		4.1	1.1	4.1	1.1	4.1	1.1
Objective	µ	394.9	338.2	293.0	282.72	346.1	291.1
	Min	208.0	155.2	251.5	247.86	163.7	111.7
	Max	1521.1	1476.1	328.5	317.64	1708.8	1748.6
	σ	108.0	110.5	9.7	9.78	130.2	133.5
	95% CI HW	2.1	2.2	0.2	0.18	2.6	2.6

## Data Availability

Not applicable.

## Supplementary Materials

The following supporting information can be downloaded at: https://www.mdpi.com/article/10.3390/ijerph192315539/s1, Figure S1: Flowchart of the studied outpatient chemotherapy process; Table S1: Appointment data of the one-week scenarios used in the experimental study; Table S2: Values of the appointment clustering features.

## Appendix A

**Table A1:** Parameters of the stochastic simulation model.

Symbol	Description
$R,R^{\prime },R^{\prime \prime }$	Number of receptionists during, before, and after regular hours of the center, respectively
G	Number of triage nurses
L	Number of lab technicians
$B,B^{\prime },B^{\prime \prime }$	Number of beds use during, before, and after regular hours of the center, respectively
C	Number of doctors during, before, and after regular working hours, respectively
$M,M^{\prime },M^{\prime \prime }$	Number of pharmacists during, before, and after regular working hours, respectively
$E,E^{\prime },E^{\prime \prime }$	Number of pharmacy technicians during, before, and after regular working hours, respectively
$A,A^{\prime },A^{\prime \prime }$	Number of pharmacy aid during, before, and after regular working hours, respectively
$N,N^{\prime },N^{\prime \prime }$	Number of nurses during, before, and after regular working hours, respectively
HDPVNC	Hour of the day when the drug orders production of advance preparations can start as per the center policy
HC	Regular center daily closing hour (the hour of the day when the center completes the regular operating time)
$XE_{p}$	Largest negative expected deviation from $x_{i}$ (from actual arrival before the scheduled time to the scheduled time) of an appointment i∈I following patient arrival distribution p
$XD_{ip}$	Dummy parameter, used to model the stochastic patients’ arrival patterns, $XD_{ip}$ equals $x_{i}-XE_{p}$ for the patient of appointment i∈I that have planned patient arrival time $x_{i}$ and following patient arrival distribution p
$XP_{ip}$	Delay time from $XD_{ip}$ to $XA_{i}$ of the patient of appointment i∈I and following arrival pattern p, where p=1 corresponds to arrival pattern of 07:00 a.m. appointments, and p=2 corresponds to arrival pattern of 11:00 a.m. appointments
$XA_{i}$	Actual patient arrival time, $XA_{i}$ equals to $XD_{ip}+XP_{ip}$ for the patient of appointment i∈I that have planned patient arrival time $x_{i}$ and following patient arrival distribution p
$DVNC_{i}$	Binary parameter, equal 1 if all the drug orders of appointment i∈I are eligible for advance preparation; 0, otherwise
BS	Percentage of patients who perform the blood test on the same day of the appointment after the registration stage
DVM	Percentage of drug orders that need modification after the drug order verification stage
DAM	Percentage of drug orders that need modifications and are modified by the doctor to keep it considered for advance preparation
DKB	Percentage of drug orders that their kits are considered to be prepared in advance
DVM	Percentage of drug orders that need modification after the drug order verification stage
$DN_{i}$	Number of drugs for the patient of appointment i∈I
$AF_{ij}$	Drug infusion duration (drug administration stage) of drug j for the patient of appointment i∈I (this parameter is deterministic because the center uses an electronic infusion pump; therefore, the stochasticity in the duration of the drug administration stage is considered in the durations of premedication AP, drug injection AI, removal AR, and observation of patient after drug infusion AO)
$LP_{u}$	Maximum acuity level that a nurse u can handle simultaneously
$LP_{i}$	Acuity level of patient of appointment i∈I
$LA_{u}$	Number of assigned patients to a nurse u at any time
RE	Registration stage delay time
T	Triage stage delay time
BE	Blood extraction delay time (blood test stage)
BT	Blood results delay time (blood test stage)
DA	Orders activation delay time
DV	Orders verification delay time (drug preparation stage)
DK	Preparation of orders kit delay time (drug preparation stage)
DP	Orders production delay time (drug preparation stage)
DC	Orders checking and bagging delay time (drug preparation stage)
DD	Orders delivery from pharmacy to bed delay time (drug preparation stage)
API	Premedication injection/preparation delay time (drug administration stage)
AP	Premedication delay time (drug administration stage)
AI	Drug injection delay time (drug administration stage)
AR	Drug removal delay time (drug administration stage)
AO	Final observation delay time (drug administration stage)
CH	Discharge stage delay time

**Table A2:** Values of the stochastic simulation model parameters used in the model validation and the experimental study.

Parameter	Value	Source
$XP_{ip}$ for p=1	exponential(67.27423, 0.97882) min, ADT *p*-value = 0.010, LRT *p*-value = 0.000	Historical Data
$XP_{ip}$ for p=2	exponential(239.49579, 0.84100) min, ADT *p*-value = 0.010, LRT *p*-value = 0.001	Historical Data
RE	weibull(1.42, 4.01, 0.90) min, ADT *p*-value = 0.016, LRT *p*-value = 0.002	Historical Data
T	weibull(1.89037, 3.96305) min, ADT *p*-value = 0.011	Historical Data
BS	7%	Historical Data
BE	triangular(2, 7, 4) min	Expert opinion
BT	triangular(5, 51, 11) min	Expert opinion & [98]
DA	weibull(2.20099, 3.99678) min, ADT *p*-value = 0.009	Historical Data
DV	$DN_{i}\times 1.5$ min	Expert opinion
DK	$DN_{i}\times 2$ min	Expert opinion
DP	$DN_{i}\times$ triangular(2, 20, 7) min	Expert opinion
DC	$DN_{i}\times 0.4$ min	Expert opinion
DD	normal(2.169, 4.141) min, ADT *p*-value = 0.025	Observation data
API	weibull(1.32, 1.2) min, ADT *p*-value = 0.014	Historical Data
AP	triangular(3, 15, 8) min	Expert opinion & observation data
AI	normal(2.13, 2.92) min	Expert opinion & [98]
AR	3.43× beta(0.67,0.577) min	Expert opinion & [98]
AO	exponential(89.70559, 0.94490) min, ADT *p*-value = 0.010, LRT *p*-value = 0.000	Historical Data
CH	weibull(1.42, 4.01, 0.90) min, ADT *p*-value = 0.016, LRT *p*-value = 0.002	Historical Data
$XE_{p}$ for p=1	45 min	Historical Data
$XE_{p}$ for p=2	267 min	Historical Data
HDPVNC	6:00 a.m.	Data
HC	3:00 p.m.	Data
$DN_{i}$	Patient-specific	Data
$DVNC_{i}$		Data
$AF_{ij}$	Patient-specific	Data
$LN_{u}$	Nurse-specific (from 4 to 6)	Data
$LP_{i}$	Patient-specific	Data

## Appendix B

**Table A3:** Results of post-optimization analysis of performance measures using Monte Carlo experiments.

Performance Measure	Average (95% CI HW)													
	Approach													
	1.1	1.2	1.3	2.1	3.1	3.2	3.3	3.4	3.5	3.6	3.7	3.8	3.9	4.1
Average patient length of stay (Makespan)	282.7 (0.2)	284.6 (0.2)	285.8 (0.2)	297.7 (0.2)	308 (0.2)	304 (0.2)	323 (0.2)	257.8 (0.1)	266.9 (0.2)	311.6 (0.2)	304.7 (0.2)	281 (0.2)	270.9 (0.2)	293 (0.2)
Average total patient waiting time	129.2 (0.2)	134.5 (0.2)	130.9 (0.2)	134 (0.2)	143.9 (0.2)	139.9 (0.2)	158.9 (0.2)	93.6 (0.1)	102.8 (0.1)	147.4 (0.2)	140.6 (0.2)	117.3 (0.2)	108.5 (0.1)	129.3 (0.2)
Average patients wait to start registration	0.6 (0)	0.6 (0)	0.6 (0)	0.7 (0)	1.7 (0)	1.7 (0)	1.7 (0)	1.7 (0)	1.7 (0)	1.7 (0)	1.7 (0)	1.8 (0)	1.7 (0)	1.4 (0)
Average patients wait to start triage	12.9 (0.1)	14.5 (0.1)	13.3 (0.1)	13.5 (0.1)	19 (0.1)	19 (0.1)	18.9 (0.1)	19 (0.1)	19 (0.1)	19 (0.1)	19 (0.1)	19 (0.1)	19 (0.1)	16.3 (0.1)
Average patients wait to start blood extraction	0 (0)	0 (0)	0 (0)	0 (0)	0 (0)	0 (0)	0 (0)	0 (0)	0 (0)	0 (0)	0 (0)	0 (0)	0 (0)	0 (0)
Average patients wait for blood test results	22.2 (0.1)	22.3 (0.1)	22.4 (0.1)	22.3 (0.1)	22.3 (0.1)	22.2 (0.1)	22.3 (0.1)	22.3 (0.1)	22.3 (0.1)	22.2 (0.1)	22.2 (0.1)	22.4 (0.1)	22.2 (0.1)	22.3 (0.1)
Average drug orders wait to start activation	2.6 (0)	2.8 (0)	2.6 (0)	1.9 (0)	3.4 (0)	3.4 (0)	2.8 (0)	2.5 (0)	2.5 (0)	2.7 (0)	2.6 (0)	2.2 (0)	2.4 (0)	2.7 (0)
Average patients wait for activation	6.2 (0)	6.4 (0)	6.2 (0)	5.5 (0)	7 (0)	6.9 (0)	6.4 (0)	6.1 (0)	6.1 (0)	6.2 (0)	6.2 (0)	5.8 (0)	5.9 (0)	6.2 (0)
Average drug orders wait to start drug order verification	23.5 (0)	27.1 (0)	23.5 (0)	11.5 (0.1)	24.5 (0)	24.5 (0)	24.5 (0)	24.4 (0)	24.4 (0)	24.5 (0)	24.5 (0)	24.4 (0)	24.4 (0)	24.4 (0)
Average drug orders wait to start preparing drug kit	1.1 (0)	1.3 (0)	1.1 (0)	0.2 (0)	1.1 (0)	1.1 (0)	1.1 (0)	1.1 (0)	1.1 (0)	1 (0)	1.1 (0)	1.1 (0)	1.1 (0)	1.1 (0)
Average drug orders wait to start drug production	91.1 (0.2)	94.7 (0.2)	92.3 (0.2)	90.9 (0.2)	99.9 (0.2)	95.9 (0.2)	114.5 (0.2)	47.6 (0.1)	57.7 (0.1)	101.9 (0.2)	95.8 (0.2)	71.6 (0.2)	61.7 (0.1)	87.4 (0.2)
Average drug orders wait to start checking and bagging	0.1 (0)	0.1 (0)	0.1 (0)	0 (0)	0.1 (0)	0.1 (0)	0 (0)	0.1 (0)	0.1 (0)	0.1 (0)	0.1 (0)	0.1 (0)	0.1 (0)	0.1 (0)
Average drug orders wait to start drug delivery	0 (0)	0.1 (0)	0 (0)	0 (0)	0 (0)	0 (0)	0.1 (0)	0 (0)	0 (0)	0 (0)	0 (0)	0 (0)	0 (0)	0 (0)
Average patients wait during drug preparation and delivery	137.8 (0.2)	141.8 (0.2)	139.3 (0.2)	128.8 (0.2)	150.1 (0.2)	145 (0.2)	165.7 (0.2)	85.3 (0.1)	97.9 (0.2)	147.6 (0.2)	143.2 (0.2)	112.6 (0.2)	100.1 (0.2)	133.9 (0.2)
Average patients wait to start premedication	0 (0)	0 (0)	0 (0)	0 (0)	0 (0)	0 (0)	0 (0)	0 (0)	0 (0)	0.1 (0)	0 (0)	0 (0)	0 (0)	0 (0)
Average patients wait to start drug injection	0 (0)	0.1 (0)	0 (0)	0.1 (0)	0 (0)	0 (0)	0.1 (0)	0 (0)	0 (0)	0.1 (0)	0.1 (0)	0 (0)	0 (0)	0 (0)
Average patients wait to start drug removal	0.1 (0)	0.1 (0)	0.1 (0)	0.1 (0)	0.1 (0)	0.1 (0)	0.2 (0)	0 (0)	0 (0)	0.2 (0)	0.1 (0)	0 (0)	0 (0)	0.1 (0)
Average patients wait to start discharge	0.1 (0)	0.1 (0)	0.1 (0)	0.1 (0)	0.1 (0)	0.1 (0)	0.2 (0)	0 (0)	0 (0)	0.2 (0)	0.1 (0)	0.1 (0)	0.1 (0)	0.1 (0)
Average patients wait after triage to start drug administration	115.6 (0.2)	119 (0.2)	116.8 (0.2)	119.6 (0.2)	123 (0.2)	118.9 (0.2)	137.8 (0.2)	72.8 (0.1)	82 (0.1)	126.2 (0.2)	119.6 (0.2)	96.3 (0.2)	87.7 (0.1)	111.4 (0.2)
Utilization beds	38.2 (0)	39.4 (0)	38.4 (0)	39.8 (0)	40.2 (0)	39.7 (0)	42.3 (0)	33 (0)	34.1 (0)	41.1 (0)	40.2 (0)	36.7 (0)	35.2 (0)	38.7 (0)
Utilization nurses	4.5 (0)	4.6 (0)	4.5 (0)	4.4 (0)	4.4 (0)	4.4 (0)	4.3 (0)	4.4 (0)	4.4 (0)	4.5 (0)	4.5 (0)	4.5 (0)	4.5 (0)	4.4 (0)
Utilization pharmacy technicians	54.4 (0)	55.2 (0)	54.3 (0)	54.9 (0)	54 (0)	53.9 (0)	54 (0)	54.7 (0)	54.4 (0)	54.3 (0)	54.2 (0)	54.5 (0)	54.8 (0)	54.2 (0)
Utilization triage nurses	23.4 (0)	24.2 (0)	23.4 (0)	23.4 (0)	23.4 (0)	23.4 (0)	23.4 (0)	23.4 (0)	23.4 (0)	23.4 (0)	23.4 (0)	23.4 (0)	23.4 (0)	23.4 (0)
Utilization of overtime receptionists before regular Hours	20.4 (0.1)	19.2 (0.1)	21 (0.1)	21.7 (0.1)	27.3 (0)	27.3 (0)	27.3 (0)	27.3 (0)	27.3 (0)	27.3 (0)	27.3 (0)	27.3 (0)	27.2 (0)	26.8 (0)
Utilization of overtime beds before regular hours	0 (0)	0 (0)	0 (0)	0 (0)	0 (0)	0 (0)	0 (0)	0 (0)	0 (0)	0 (0)	0 (0)	0 (0)	0 (0)	0 (0)
Utilization of overtime nurses before regular hours	0 (0)	0 (0)	0 (0)	0 (0)	0 (0)	0 (0)	0 (0)	0 (0)	0 (0)	0 (0)	0 (0)	0 (0)	0 (0)	0 (0)
Utilization of overtime pharmacists before regular hours	6.7 (0)	6.5 (0)	6.3 (0)	4 (0)	6.7 (0)	7.4 (0)	7.8 (0)	5.9 (0)	5.9 (0)	7.6 (0)	6.3 (0)	6.4 (0)	5.8 (0)	7 (0)
Utilization of overtime drug deliverers before regular hours	331.3 (2.7)	357.8 (2.6)	321.6 (2.6)	164.4 (2.3)	361.3 (2.9)	369.7 (2.9)	327.6 (2.6)	335.7 (2.7)	347.1 (2.8)	303.6 (2.5)	325.9 (2.6)	292 (2.5)	281.1 (2.3)	338.2 (2.8)
Utilization of overtime pharmacy technicians before regular hours	9.1 (0.1)	10.7 (0.1)	10 (0.1)	4.9 (0.1)	12.3 (0.1)	12.4 (0.1)	11.5 (0.1)	7.1 (0.1)	9 (0.1)	9.7 (0.1)	10.7 (0.1)	8.4 (0.1)	5.5 (0)	11.1 (0.1)
Utilization of overtime receptionists after regular Hours	0 (0)	0 (0)	0 (0)	0 (0)	0 (0)	0 (0)	0 (0)	0 (0)	0 (0)	0 (0)	0 (0)	0 (0)	0 (0)	0 (0)
Utilization of overtime beds after regular hours	0 (0)	0 (0)	0 (0)	0 (0)	0 (0)	0 (0)	0 (0)	0 (0)	0 (0)	0 (0)	0 (0)	0 (0)	0 (0)	0 (0)
Utilization of overtime nurses after regular hours	3.6 (0)	3.9 (0)	3.7 (0)	4.2 (0)	4.1 (0)	3.9 (0)	4.2 (0)	3.8 (0)	4.2 (0)	3.5 (0)	3.5 (0)	3.7 (0)	3.9 (0)	4 (0)
Utilization of pharmacists after regular hours	0 (0)	0.1 (0)	0 (0)	0.1 (0)	0 (0)	0 (0)	0 (0)	0.1 (0)	0.1 (0)	0 (0)	0 (0)	0.1 (0)	0.1 (0)	0 (0)
Utilization of overtime drug deliverers after regular Hours	24.5 (0.4)	36.2 (0.5)	24.9 (0.4)	27.2 (0.5)	25.9 (0.4)	24.1 (0.4)	38.6 (0.7)	20.7 (0.3)	20.3 (0.3)	31.6 (0.5)	24 (0.4)	23.6 (0.4)	24.7 (0.4)	24.8 (0.4)
Utilization of overtime pharmacy technicians after regular hours	0.4 (0)	0.6 (0)	0.4 (0)	0.4 (0)	0.5 (0)	0.4 (0)	0.6 (0)	0.3 (0)	0.3 (0)	0.5 (0)	0.4 (0)	0.4 (0)	0.4 (0)	0.4 (0)
Number of drug order verifications before patient arrival	88.6 (0)	87.6 (0)	88.6 (0)	88.6 (0.1)	88.6 (0)	88.6 (0)	88.6 (0)	88.6 (0)	88.6 (0)	88.6 (0)	88.6 (0)	88.6 (0)	88.6 (0)	88.6 (0)
Number of drug order kits prepared before patient arrival	88.6 (0)	87.6 (0)	88.6 (0)	88.6 (0.1)	88.6 (0)	88.6 (0)	88.6 (0)	88.6 (0)	88.6 (0)	88.6 (0)	88.6 (0)	88.6 (0)	88.6 (0)	88.6 (0)
Number of ready kits of eligible drug orders for advance production before the specified time to start advance drug preparations by the center policy (6:00 a.m.)	43.9 (0)	41.9 (0)	43.9 (0)	44.1 (0.1)	43.9 (0)	43.9 (0)	43.9 (0)	43.9 (0)	43.9 (0)	43.9 (0)	43.9 (0)	43.9 (0)	43.9 (0)	43.9 (0)
Mean overtime of receptionists	0.2 (0)	0.2 (0)	0.2 (0)	0.2 (0)	0.2 (0)	0.2 (0)	0.2 (0)	0.2 (0)	0.2 (0.1)	0.2 (0)	0.2 (0)	0.2 (0.1)	0.2 (0)	0.2 (0)
Mean overtime of pharmacy technicians	11.7 (0.6)	15 (0.6)	11.2 (0.6)	12.3 (0.6)	12.3 (0.6)	12.6 (0.6)	12.9 (0.6)	13 (0.6)	13.1 (0.6)	12.7 (0.6)	12.2 (0.6)	12.9 (0.6)	13.3 (0.6)	12 (0.6)
Mean overtime of pharmacists	11.9 (0.6)	15.3 (0.6)	11.5 (0.6)	12.6 (0.6)	12.5 (0.6)	12.8 (0.6)	13 (0.6)	13.5 (0.6)	13.5 (0.6)	12.8 (0.6)	12.4 (0.6)	13.2 (0.6)	13.7 (0.6)	12.2 (0.6)
Mean overtime of nurses	267.3 (1.4)	280.3 (1.4)	277.8 (1.4)	317.2 (1.3)	349.5 (1.3)	347.4 (1.3)	328.4 (1.4)	340.2 (1.3)	350.3 (1.3)	305.7 (1.4)	308.2 (1.4)	322.4 (1.3)	324.2 (1.4)	321.7 (1.3)
