# Supplementary material for: Clustering and Stochastic Simulation Optimization for Outpatient Chemotherapy Appointment Planning and Scheduling
Source: Int J Environ Res Public Health. 2022 Nov 23;19(23):15539. doi: 10.3390/ijerph192315539 (PMC9736607; doi:10.3390/ijerph192315539)
Supplement: Supplementary file 1 [file ijerph-19-15539-s001.zip › Table S2. Values of the appointment clustering features.pdf]

appointment unique identifier	target treatment day	total infusion duration for all drugs	number of drugs to be prepared	all drugs eligible for advance preparation
1	1	60	1	1
2	1	210	2	1
3	1	105	2	1
4	1	60	2	0
5	1	70	2	1
6	1	210	2	1
7	1	30	2	0
8	1	90	1	1
9	1	70	1	1
10	1	0	1	0
11	1	30	1	0
12	1	30	2	0
13	1	60	1	1
14	1	30	1	0
15	1	90	4	0
16	1	0	1	1
17	1	0	1	1
18	1	150	3	0
19	1	130	2	1
20	1	60	1	1
21	1	120	2	0
22	1	210	3	0
23	1	60	1	1
24	1	60	1	1
25	1	0	1	0
26	1	94	3	0
27	1	30	1	0
28	1	30	1	0
29	1	215	1	1
30	1	150	2	1
31	1	150	3	0
32	1	30	1	1
33	1	15	2	1
34	1	30	1	1
35	1	30	1	0
36	1	10	1	1
37	1	90	2	1
38	1	60	2	0
39	1	30	1	0
40	1	90	2	1
41	1	0	2	0
42	1	120	1	1
43	1	0	1	0
44	1	30	2	1
45	1	60	1	1
46	1	60	1	1
47	1	90	2	1
48	1	30	2	0
49	1	75	3	1
50	1	120	1	1
51	1	90	4	0
52	1	150	3	0
53	1	10	1	1
54	1	200	1	1
55	2	360	7	0
56	2	105	2	1
57	2	30	1	1
58	2	210	2	1
59	2	30	1	1
60	2	30	1	0
61	2	0	1	1
62	2	80	1	1
63	2	60	1	1
64	2	30	2	0
65	2	30	2	0
66	2	10	2	0
67	2	90	1	1
68	2	100	2	1
69	2	150	2	1
70	2	90	4	0
71	2	270	4	0
72	2	30	2	0
73	2	90	4	0
74	2	120	3	0
75	2	30	1	0
76	2	90	2	1
77	2	210	2	1
78	2	90	4	0
79	2	90	2	0
80	2	100	2	1
81	2	60	1	1
82	2	120	1	1
83	2	240	4	0
84	2	0	1	1
85	2	0	1	0
86	2	30	1	0
87	2	77	2	1
88	2	60	1	1
89	2	30	1	1
90	2	60	1	1
91	2	60	1	1
92	2	60	2	1
93	2	130	4	0
94	2	60	1	1
95	2	210	4	0
96	2	100	1	1
97	2	6	1	1
98	2	30	1	1
99	3	105	2	1
100	3	30	2	0
101	3	0	1	1
102	3	150	2	1
103	3	120	2	1
104	3	60	2	0
105	3	30	1	1
106	3	60	1	1
107	3	160	4	0
108	3	180	3	0
109	3	160	4	0
110	3	60	1	0
111	3	0	2	0
112	3	60	2	0
113	3	150	2	0
114	3	170	2	1
115	3	60	2	0
116	3	60	1	1
117	3	60	2	0
118	3	75	3	1
119	3	30	2	0
120	3	90	2	1
121	3	60	2	1
122	3	130	2	1
123	3	0	1	0
124	3	60	1	0
125	3	60	1	1
126	3	70	1	1
127	3	120	1	1
128	3	120	2	0
129	3	90	1	1
130	3	60	1	1
131	3	30	2	0
132	3	120	1	1
133	3	120	1	1
134	3	30	2	0
135	3	90	1	0
136	3	90	2	1
137	3	60	1	0
138	3	60	1	1
139	3	70	1	1
140	4	165	4	0
141	4	60	1	1
142	4	60	2	0
143	4	100	2	1
144	4	360	5	1
145	4	150	2	0
146	4	30	1	0
147	4	0	1	0
148	4	60	2	0
149	4	0	1	0
150	4	60	1	1
151	4	10	1	1
152	4	10	1	0
153	4	60	2	0
154	4	0	1	0
155	4	210	3	0
156	4	30	2	0
157	4	60	1	0
158	4	60	1	1
159	4	75	2	1
160	4	0	1	1
161	4	60	1	0
162	4	180	3	0
163	4	60	1	1
164	4	90	4	0
165	4	75	2	1
166	4	0	1	0
167	4	90	2	1
168	4	120	1	1
169	4	25	2	1
170	4	0	1	0
171	4	0	1	0
172	4	60	1	1
173	4	60	1	0
174	4	90	2	1
175	4	150	2	0
176	4	60	1	1
177	4	30	2	0
178	4	30	1	1
179	4	160	4	0
180	4	0	1	0
181	4	60	1	1
182	4	30	1	1
183	4	60	1	0
184	4	60	1	1
185	4	0	1	0
186	4	30	2	0
187	4	30	1	1
188	4	30	1	1
189	4	75	4	1
190	4	60	1	1
191	5	600	7	0
192	5	60	1	1
193	5	30	1	0
194	5	60	1	1
195	5	30	1	0
196	5	120	1	1
197	5	30	2	0
198	5	90	5	0
199	5	150	2	0
200	5	30	1	0
201	5	10	1	1
202	5	60	1	0
203	5	60	1	1
204	5	30	1	0
205	5	30	1	0
206	5	70	3	0
207	5	30	1	0
208	5	30	1	0
209	5	0	1	1
210	5	90	4	0
211	5	30	1	1
212	5	130	4	0
213	5	30	2	0
214	5	60	1	1
215	5	30	1	0
216	5	0	1	1
217	5	15	2	1
218	5	60	1	1
219	5	90	4	0
220	5	10	2	0
221	5	10	1	0
222	5	40	2	1
223	5	60	2	1
224	5	120	1	1
225	5	130	2	1
226	5	60	2	0
227	5	60	2	0
228	5	60	1	1
229	5	60	1	1
230	5	60	1	1
231	5	90	1	0
232	5	30	2	0
233	5	0	1	0
234	5	90	1	1
235	5	60	1	1
236	5	0	1	0
237	5	60	2	0
238	5	120	1	1
239	5	150	3	0
240	5	90	2	1
241	5	130	2	1
242	5	60	1	1
243	5	30	1	1
244	5	0	1	0
245	5	130	2	1
246	5	120	2	1
