# Supplementary figures and images for: Clustering and Stochastic Simulation Optimization for Outpatient Chemotherapy Appointment Planning and Scheduling
Source: Int J Environ Res Public Health. 2022 Nov 23;19(23):15539. doi: 10.3390/ijerph192315539 (PMC9736607; doi:10.3390/ijerph192315539)

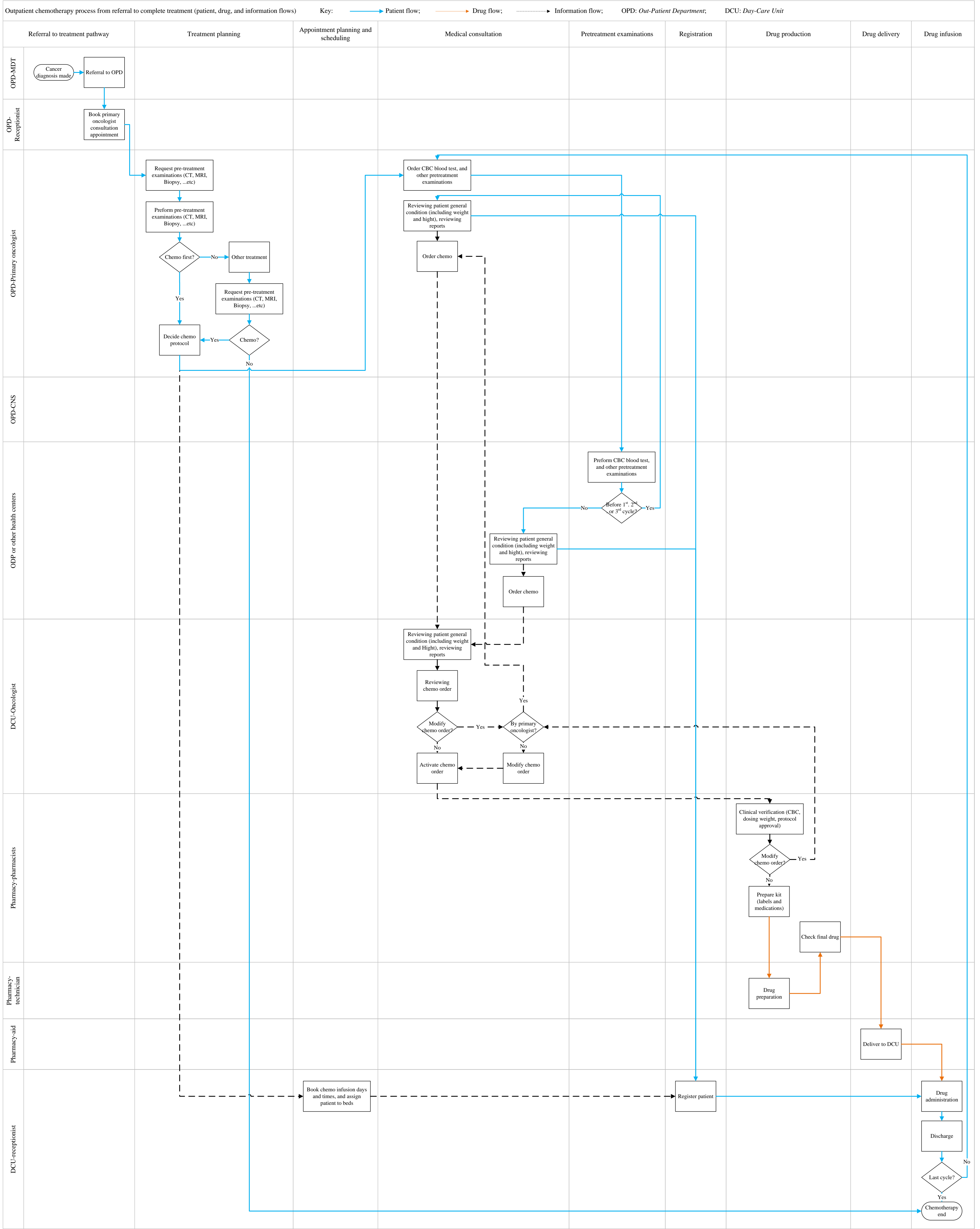

Supplement: Supplementary file 1 [file ijerph-19-15539-s001.zip › Figure S1. Flowchart of the studied outpatient chemotherapy process.pdf]
